# Safer-by-design flame-sprayed silicon dioxide nanoparticles: the role of silanol content on ROS generation, surface activity and cytotoxicity

**DOI:** 10.1186/s12989-019-0325-1

**Published:** 2019-10-29

**Authors:** Laura Rubio, Georgios Pyrgiotakis, Juan Beltran-Huarac, Yipei Zhang, Joshi Gaurav, Glen Deloid, Anastasia Spyrogianni, Kristopher A. Sarosiek, Dhimiter Bello, Philip Demokritou

**Affiliations:** 1 Center for Nanotechnology and Nanotoxicology, HSPH-NIEHS Nanosafety Center, Department of Environmental Health, Harvard T. H. Chan School of Public School, Harvard University, 665 Huntington, Boston, MA 02115 USA; 2 Department of Biomedical and Nutritional Sciences, Zuckerberg College of Health Sciences, University of Massachusetts Lowell, Lowell, MA 01854 USA; 3 John B. Little Center for Radiation Sciences, Department of Environmental Health, Harvard T.H. Chan School of Public Health, Boston, MA 02115 USA; 4 Particle Technology Laboratory, Institute of Process Engineering, Department of Mechanical and Process Engineering, ETH Zurich, Sonneggstrasse 3, CH-8092 Zurich, Switzerland

**Keywords:** Silanol groups, Amorphous silica, Surface reactivity, Flame spray pyrolysis, Toxicity

## Abstract

**Background:**

Amorphous silica nanoparticles (SiO2 NPs) have been regarded as relatively benign nanomaterials, however, this widely held opinion has been questioned in recent years by several reports on in vitro and in vivo toxicity. Surface chemistry, more specifically the surface silanol content, has been identified as an important toxicity modulator for SiO2 NPs. Here, quantitative relationships between the silanol content on SiO_2_ NPs, free radical generation and toxicity have been identified, with the purpose of synthesizing safer-by-design fumed silica nanoparticles.

**Results:**

Consistent and statistically significant trends were seen between the total silanol content, cell membrane damage, and cell viability, but not with intracellular reactive oxygen species (ROS), in the macrophages RAW264.7. SiO_2_ NPs with lower total silanol content exhibited larger adverse cellular effects. The SAEC epithelial cell line did not show any sign of toxicity by any of the nanoparticles. Free radical generation and surface reactivity of these nanoparticles were also influenced by the temperature of combustion and total silanol content.

**Conclusion:**

Surface silanol content plays an important role in cellular toxicity and surface reactivity, although it might not be the sole factor influencing fumed silica NP toxicity. It was demonstrated that synthesis conditions for SiO_2_ NPs influence the type and quantity of free radicals, oxidative stress, nanoparticle interaction with the biological milieu they come in contact with, and determine the specific mechanisms of toxicity. We demonstrate here that it is possible to produce much less toxic fumed silicas by modulating the synthesis conditions.

## Background

Amorphous silica (SiO2) nanoparticles (NPs) are among the most widely produced engineered nanomaterials (ENMs), second only to carbon black [1], and used in a myriad of applications, owing to their large surface area, tunable surface properties, chemical and thermal stability, mechanical robustness, and low production costs [2]. These applications include filler agents, absorbents, catalysts, toners and inorganic carriers in fields comprising biomedicine, cosmetics, food industry, and printing equipment, among many others [3–7].

SiO_2_ is produced by wet (e.g. Stöber) [8] or gas-phase methods (e.g. in vapor-fed flame reactors or by flame spray pyrolysis - FSP) [9]. Flame synthesis is one of the most common large-volume methods by which millions of tons of SiO_2_ NPs (also referred to as fumed or pyrogenic silica) are produced annually [9]. One important advantage of FSP is scalability, enabling large scale manufacturing of nanoparticles, which makes it preferable over conventional methods such as wet-chemistry technologies [10, 11].

Although this ENM is classified as Generally Regarded as Safe (GRAS) by the Food and Drug Administration (FDA), and used extensively in consumer products, including as a food additive in numerous food products [12], this assumption has been questioned in recent years in light of several new reports on the toxicity of silica, in vitro and in vivo [13–20]. Commonly reported cellular effects of fumed silica in vitro, include ROS production, lipid peroxidation, pro-inflammatory responses, DNA damage, and loss of membrane integrity [13, 21–24]. In in vivo studies, pro-inflammatory effects were documented in the lung of Wistar rats following acute exposures [25], whilst sub-acute and sub-chronic ingestion and intravenous exposures caused fibrosis in kidney and spleen [26, 27]. In light of these new findings, a re-evaluation of the safety and/or potentially harmful effects from chronic exposures to amorphous silica is warranted.

Several studies have shown that surface chemistry and reactivity of SiO_2_ NPs play an important role in cellular responses and toxicity [18–20]. The synthesis method regulates the surface chemistry by modulating the number of silanol groups (−Si-O-H), which in turn modulates the toxicity of the amorphous SiO_2_ NPs [28]. However, the exact molecular mechanisms involved in this toxicity and the role of silanol groups remain uncertain. Studies have shown surface silanol content of SiO2 NPs to cause cellular toxicity and cell damage through the production of reactive oxygen species (ROS) and ensuing oxidative stress [20]. SiO_2_ NPs have also been shown to cause hemolysis [14, 20, 28–31]. Zhang et al. have proposed a conformation of siloxane present in fumed SiO_2_ NPs, the 3-member siloxane rings (3MRs), as precursors of ROS. These reactive molecules would oxidize the polyunsaturated fatty acids in cell membranes, triggering production of other signaling molecules (such as 8-isoprostane) and leukotrienes, deplete intracellular antioxidants, cause damage to DNA/nucleic acids, and proteins, interfere with other normal cellular biological processes, which, in turn, may lead to inflammation an/or cell death. However, there is no agreement on this point, since other studies have shown ROS-independent pathways linked to fumed SiO_2_ NPs exposures, including membranolysis [32–34].

It is generally postulated that SiO_2_ NPs synthesized via wet chemistry methods contain significantly higher surface silanol density compared to the fumed silicas produced by flame pyrolysis, presumably due to the water matrix that can react with the SiO_2_ surface [35, 36]. On the contrary, during the synthesis of fumed silica, the high temperature favors the conversion of ≡Si-O-H to ≡Si-O-Si≡, a conversion that depends on the flame temperature [37]. Therefore, it is possible, in theory, to finely tune the surface chemistry of the SiO_2_ NPs and modify the silanol content, by modulating the combustion enthalpy during their synthesis, thus mitigating potential bioactivity [18]. Although several studies have already investigated this effect, it is unclear whether or not the silanol content can affect the final toxicity outcome. More importantly, most of these studies have overlooked the cellular dosimetry of silica, how the particle properties can influence agglomeration in culture media and particle kinetics, which can affect the effective dose, delivered-to cells. In previous studies, it was demonstrated that SiO_2_ NPs, in particular, have very low delivered-to cell-doses and that silicas with different surface chemistries can result in different delivered dose rates [38–40]. Further, it has been shown that, when taking into consideration the dosimetry and delivered doses and dose rates, the hazard ranking of ENMs can change [41].

In this study, different fumed SiO2 NPs with different silanol content were synthesized and used to assess the effect of the silanol content on cellular toxicity. The silanol groups were modulated by adjusting the enthalpy of the flame according to the work recently published by the authors [18]. In this study, we took into consideration the differential settling rates of various SiO2 NPs, which may differ due to different surface chemistries, and incorporated them into dose-response analysis using delivered doses instead of administered doses. These doses were chosen over a broad range of silica concentrations in order to observe biological differences among different SiO2 NPs, and do not necessarily reflect environmental exposures. This study provides insights into the effects of gradual changes in silanol surface content on SiO_2_ NPs ROS production, surface reactivity, necrotic potency (membrane disruption) and biological activity resulting in increased cellular toxicity. We also demonstrate that safer materials can be produced by modulating the flame synthesis conditions (enthalpy content) of fumed SiO_2_ NPs for various consumer products.

## Methods

### Synthesis of SiO_2_ NPs

A panel of five SiO_2_ NPs particles was produced by FSP as previously reported in the literature [18, 42]. Briefly, a precursor solution of hexamethyldisiloxane (HMDSO, puriss, #98.5%) in ethanol (puriss, #98.5%) at various Si concentration was prepared. The solution was fed with a syringe pump in a metal capillary with a predetermined rate (*x* ml/min) where it was dispersed by an oxygen flow (*y* l/min) that had a 1.5 bar pressure drop at the nozzle tip. The aerosolized precursor was then ignited with a supporting flame of premixed 1.5 L/min CH_4_/3.2 L/min O_2_. The produced heat converts the precursor to the metal oxide. The ratio *x/y* determines physicochemical properties such as the primary particle size and silanol content.

In addition commercially available fumed SiO_2_ NPs (Aerosil® 200) and WetChem SiO_2_ NPs (porous SiO_2_ NPs mostly synthesized by wet-chemistry approaches) were obtained as benchmark materials from Evonik Industries and Sigma Aldrich (product # 637246), respectively, and were hereafter denoted as Commercial fumed SiO_2_ and WetChem SiO_2_.

#### Enthalpy estimation for FSP-made SiO_2_ NPs

For each flame the ratio of the combustion enthalpy introduced by precursor, solvent and methane into the flame ($\dot{\mathrm{H}}$_C_ in MJ/min) over the total (liquid and gas) inlet mass flow rate, m_tot,in_ (kg/min) was calculated, based on Spyrogianni et al. [18] as follows:

$$ \frac{{\dot{\mathrm{H}}}_{\mathrm{C}}}{{\dot{\mathrm{m}}}_{\mathrm{tot},\mathrm{in}}}=-\frac{\left({\dot{\mathrm{n}}}_{\mathrm{precursor}}\Delta {\mathrm{H}}_{\mathrm{C}}^{\mathrm{precursor}}+{\dot{\mathrm{n}}}_{\mathrm{solvent}}\Delta {\mathrm{H}}_{\mathrm{C}}^{\mathrm{solvent}}+{\dot{\mathrm{n}}}_{\mathrm{C}{\mathrm{H}}_4}\Delta {\mathrm{H}}_{\mathrm{C}}^{\mathrm{C}{\mathrm{H}}_4}\right)}{{\dot{\mathrm{m}}}_{\mathrm{precursor}-\mathrm{solution}+}{\dot{\mathrm{m}}}_{\mathrm{dispersion}-{\mathrm{O}}_2+}{\dot{\mathrm{m}}}_{\mathrm{flame}-{\mathrm{C}\mathrm{H}}_4+}{\dot{\mathrm{m}}}_{\mathrm{flame}-{\mathrm{O}}_2}} $$

where $\dot{\mathrm{n}}$ and $\dot{\mathrm{m}}$ are the inlet flow rates in mol/min and kg/min, respectively, and ΔH_C_ (MJ/mol) is the combustion enthalpy of each compound. ΔH_C_ was calculated assuming complete combustion using the reactant and product enthalpies of formation (at 25 °C) from Iseard et al. [43] for HMDSO and from Haynes [44] for ethanol, CH_4_, CO_2_ and H_2_O. SiO_2_ NPs from low enthalpy flames (< 11 MJ/kg) are referred to as “cold SiO_2_ NPs” (FSP SiO_2_ 5/5, FSP SiO_2_ 3/5, FSP SiO_2_ 4/5) while high enthalpy (> 15 MJ/kg) SiO_2_ NPs are reported as “hot SiO_2_ NPs” (FSP SiO_2_ 9/3, FSP SiO_2_ 11/3, commercial fumed SiO_2_). The commercial WetChem SiO_2_ NPs is considered as cold silica since there is no heat production in the synthesis process.

### Physicochemical characterization of SiO_2_ NPs

#### Tem

Transmission electron microscopy (TEM, FEI Tecnai F30 ST microscope operated at 300 kV) was used to ascertain the morphology of different types of SiO_2_ NPs. The powders were dispersed in ethanol at 100 μg/ml with cup-horn sonication at 100 kJ/L energy (95% amplitude, 30s on, 1 s off) [18] and deposited onto a perforated carbon foil supported by a copper grid. A similar sample preparation procedure was used to analyze the morphology of FSP SiO_2_ 5/5 via a TEM, JEOL 2100 [42].

#### Bet

Brunauer-Emmett-Teller (BET) N_2_-adsorption at 77 K was conducted to determine the specific surface area (SSA) of SiO_2_ NPs using a five-point BET isotherm ((TriStar II Plus, Micromeritics) after degassing the samples for ≥1 h at 140 °C) [10]. Similar protocols were used to determine the SSA of FSP SiO_2_ 5/5 via a high-throughput surface area and pore-size analyzer (Quantachrome Instruments, NOVAtouch LX^4^). The equivalent BET particle size (*d*_BET_) was calculated assuming that the particles are spherical and of equal size, and following *d*_BET_ = 6000/(SSA·*ρ*), where the SSA is the specific surface in m^2^/g, *ρ* is the material density in g/cm^3^, and *d*_BET_ is in nm [42].

#### Density

SiO_2_ NPs density in powder form was measured using a pycnometer (Quantachrome Instruments, ULTRAPYC 1200e). SiO_2_ NPs in powder form were used without any preparation. The sample volume was measured 15 times and the average value was used as the value of the density.

#### Silanol content

The total silanol content (surface and internal) of all samples but the FSP 5/5 SiO_2_ has been previously reported [18]. For the FSP 5/5 SiO_2_ NPs, the total silanol content was quantified by thermogravimetric analysis (TGA) with a thermobalance (TGA/SDTA 851e, Mettler Toledo), modifying a previously described procedure [18]. Briefly, slightly compacted SiO_2_ powder was filled into 900 μL alumina crucibles and a TGA method consisting of two steps was employed. In step 1, the samples were heated in 40 ml/min Ar from 40 to 140 °C at 5 °C/min and held at this temperature for 180 min. In step 2, the gas flow was changed to 40 ml/min O_2_ to allow for the oxidation of possible carbon-containing residues on the particle surface. The temperature was increased to 800 °C at 10 °C/min and held constant for 60 min. The mass loss during step 2 was used to calculate the number of silanol groups per surface area according to:

$$ \mathrm{OH}/{\mathrm{nm}}^2=2\left[\left({\mathrm{m}}_{140{}^{\circ}\mathrm{C}}-{\mathrm{m}}_{800{}^{\circ}\mathrm{C}}\right)\ {\mathrm{N}}_{\mathrm{A}}/{\mathrm{M}}_{\mathrm{H}2\mathrm{O}}\right]/\left[{10}^{18}\ \mathrm{SSA}\ {\mathrm{m}}_{140{}^{\circ}\mathrm{C}}\ \right]+1 $$

where m_140 °C_ and m_800 °C_ are the sample mass in grams at the beginning and at the end of step 2, SSA is in m^2^/g, M_H2O_ (g/mol) is the molar mass of H_2_O and N_A_ (#/mol) is Avogadro’s constant [45–48]. Furthermore, it is assumed that the silica surface is still covered with one hydroxyl group per nm^2^ at 800 °C [49].

#### X-ray photoelectron spectroscopy

XPS was used to analyze the surface chemistry of the SiO_2_ NPs (quantification of the ≡Si–O–H, −O–Si–O–, and organic carbon/oxygen) and its stoichiometry. The NPs powders were used without any treatment. A double-sided carbon tape was used to fix the powders on the XPS plate. Approximately 100 mg of the ENM were pressed to create a small pellet that was placed on the carbon tape and pressed to adhere on the carbon tape. The Thermo Scientific K-Alpha XPS was used to perform XPS analysis. The survey range was set from − 10 eV to 1350 eV with 200 eV pass energy and 400 μm spot size. Three different spots of the pellet were used for the survey. Once all the elements were identified, high-resolution elemental scans were performed for each element to minimize the Noise to Signal ratio (N2S) without saturating the detector. For the data analysis, the calculation software Avantage™ Software (Thermo Scientific, Waltham, MA) was used. The XPS spectra were calibrated in respect to the Carbon 284.6 eV peak.

### Suspension preparation, colloidal characterization and dosimetry analysis of SiO_2_ NPs for cellular studies

#### Suspension preparation and colloidal characterization

The dispersion preparation, colloidal characterization and dosimetric analysis were performed as described in great detail by the authors in previous publications [40, 42, 50, 51]. The cup horn sonicator (Branson Sonifier S-450D, 400 W, with Branson 3-in. cup horn) was calibrated according to the protocol by Taurozzi et al. [51] and found to deliver 2.59 W/ml. A stock solution of ENMs in distilled water (Invitrogen) was prepared at a concentration of 0.5 mg/ml and was used to determine the critical delivered sonication energy (DSE_cr_). One milliter of the stock solution was used to measure the hydrodynamic diameter (dH) with DLS (Malvern Nanosizer, Worcestershire, UK). The solution was sonicated for 1 min, vortexed for 30 s, and measured again. The process continued until the dH and polydispersity index (PDI) were not changing significantly (± 5%). The DSE_cr_ of an ENM is defined as the DSE (in J/ml) required to achieve the lowest particle agglomeration state in DI H_2_O and is ENM-specific. Once the DSE_crt_ was determined, a fresh suspension was prepared, and it was diluted with RPMI+ 10% (vol/vol) FBS to a final concentration of 0.1 mg/ml and its dH was measured with DLS at 0 h and 24 h to assess stability overtime. Further, the effective density (ρ_eff_) was determined using the volumetric centrifugation method (VCM) as described previously [42].

#### Fate and transport modeling for calculation of dose delivered to cells

The distorted grid (DG) model was utilized to calculate the concentration profiles across the well of a 96-well plate, the concentration at the bottom of the well (bottom concentration) and the fraction of deposited particles to the cell surface as a function of the exposure time (f_D_) for the SiO_2_ NP suspensions [51]. The developed code was executed on MATLAB (MathWorks, Massachusetts, USA). Inputs for the model were the agglomerate volume-weighted hydrodynamic diameter (d_H_) and ρ _eff_ of SiO_2_ NPs agglomerates suspended in RPMI+ 10% (vol/vol) FBS.

### Acellular ROS characterization of SiO_2_ NPs

Acellular measurement of ROS generated by various silica NPs is based on the oxidation of Trolox (a water-soluble variant of Vitamin E) to Trolox quinone (TQ), followed by TQ quantitation with liquid chromatography-electrospray ionization-tandem mass spectrometry (LC-ESI-MS/MS). The principle of the method is described in Zhao et al. [52]. The method quantifies the highly reactive radicals (such as hydroxyl radicals, superoxide anions, and singlet oxygen), as a group (fast-reacting ROS) as well as the stable hydrogen peroxide (H_2_O_2_) [53]. Notable advantages of this method over traditional fluorescin-based assays such as the DCFH assay are high sensitivity, interference-free measurements, and simultaneous quantitation of fast-reacting ROS and H_2_O_2_.

#### Incubation of SiO_2_ NPs with Trolox

Different volumes of 1 mg/ml stock of SiO2 NPs (10, 25, 50, 100 μL) were added to 0.5 ml solution (containing 100 nmol) Trolox in 7 ml amber vials, and the volume was adjusted to a final 1 ml with phosphate buffer (50 μM, pH 7.4), to obtain a range of SiO2 NPs concentrations (10 μg/ml – 100 μg/ml) in 0.1 mM Trolox solution in pH 7.4 phosphate buffer. The above vials were placed in a Thermo Forma 420 shaker (Thermo Fisher Scientific, Waltham, MA, USA) at 37 °C and 50 rpm for 30 min. Then the samples were filtered through a 20 nm pore PTFE membrane filter (Whatman, 10 mm diameter) to remove SiO2 NPs. NP removal efficiency was confirmed via DLS and Tunable Resistive Pulse Sensing (TRPS) measurements of the filtrate showing no particles present. Two 0.4 ml aliquots of the filtrate Trolox solution were transferred into two separate 1.8 ml amber liquid chromatography vials. One unit of horseradish peroxidase (HRP) was added into one of the vials used for H_2_O_2_ quantification. The other vial was not modified. Subsequently, both vials were incubated at 37 °C for 30 min and then subjected to LC-ESI–MS/MS analysis for Trolox Quinone (TQ) quantitation.

#### LC-ESI-MS/MS analysis

Trolox and TQ, were analyzed by LC–ESI-MS/MS as described earlier [52, 54]. Electrospray ionization (ESI) was performed in the positive ion mode (ion spray voltage 5000 V) with nitrogen as the nebulizing, heater, curtain, and collision gas. Gas flow parameters were optimized (nebulizer 65 psi, heater 50 psi, and curtain gas 30 psi) by making successive flow injections while introducing mobile phase into the ionization source at 600 μL/min. The turbo ion spray temperature was set to 500 °C. Quantitative analysis was performed in the multiple reaction monitoring (MRM) mode by monitoring the transition 267➔221, with a dwell time of 500 ms. The following compound-specific parameters used were: Declustering potential DP, 48 V; collision energy, 21 (eV); and collision energy exit potential, 5. Chromatographic separation was achieved on a Kinetex C18 column, (4.6 × 100 mm, 2.6 μm particle size) (Phenomenex, Torrance, CA) at a flow rate of 600 μL/min and column temperature set at 40 °C. The isocratic separation was accomplished with 60% solvent A (0.1% ammonium acetate in water) and 40% solvent B (0.1% formic acid in methanol). Injection volume was 10 μL.

#### Fast reacting ROS and H_2_O_2_ quantitation

Fast reacting ROS species (such as hydroxyl and superoxide radicals), which have a short half-life in the milliseconds’ range, were measured as the amount of TQ formed in the first vial [52]. H_2_O_2_, a stable product, does not react appreciably with Trolox under the current experimental conditions (verified experimentally), but in the presence of HRP it is converted to hydroxyl radical (1H_2_O_2_:1OH•) which oxidizes Trolox to TQ. The amount of TQ in vial #2 (with HRP) is the sum of fast-reacting ROS species and H_2_O_2_. H_2_O_2_ was calculated from the difference between TQ in the HRP containing vial (#2, total ROS) and TQ in vial #1 (fast-reacting ROS). This approach was validated independently by treating H_2_O_2_ containing samples and standards with catalase, an enzyme specialized in converting H_2_O_2_ into water, and then re-quantifying ROS. In the presence of catalase, the amount of H_2_O_2_ was reduced to zero.

### In-vitro cellular studies

#### Cell culture

RAW 264.7 cells, purchased from ATCC (ATCC, Rockville, MD), were grown as a monolayer using DMEM medium (Gibco-Life Technologies) supplemented with 10% heat-inactivated FBS, 100 IU/ml Penicillin, 100 μg/ml Streptomycin (Gibco-Life Technologies) and 1 mM HEPES (Gibco-Life Technologies). Normal small airway epithelial cells (SAEC) were purchased from Lonza (Walkersville, Maryland) and maintained in serum-free SABM with the following supplemental growth factors (Bovine Pituitary Extract, Hydrocortisone, Human Epidermal Growth Factor, Epinephrine, Transferrin, Insulin, Retinoic, Triiodothyronine, Gentamicin Amphotericin-B, and BSA-fatty acid free) provided by the manufacturer (Lonza Inc., Allendale, New Jersey). Both cell lines were cultured at 37 °C in a humidified 5% CO2 incubator and subcultured at 80% confluence.

#### Cellular treatment

For each experiment, cells were plated at 50,000 cells/well in a 96-well plate and allowed to fully attach for 24 h. After that, the medium was changed to RPMI 10% FBS and cells were treated with the different SiO2 NPs. Based on the dosimetry data obtained via the DG model [40, 51], the administered doses were chosen to yield the delivered-to-cell dose in terms of mass per surface (μg/cm^2^). The delivered-to-cell doses for all the materials at 24 h were 0.026, 0.052 and 0.104 μg/cm^2^. After 24 h of treatment, cells were analyzed for different toxicological endpoints.

#### Cellular membrane integrity

After being exposed to the test particles for 24 h, cells were evaluated for cytotoxicity using the Pierce™ LDH Cytotoxicity Assay Kit (Thermo Scientific, Waltham, MA, USA). LDH release, used as an indicator of cell membrane damage, was measured in the culture medium according to the manufacturer’s instruction. Briefly, 80 μl cell-free supernatants from the culture treatments were collected, and centrifuged at 2000 rpm for 10 min; 50 μl of the media supernatant was then added to a fresh 96-well plate along with LDH assay mix reagent. After incubating for 30 min, the absorbance values were recorded at both 490 nm and 680 nm using SpectraMax M5/M5e (Molecular Devices, Sunnyvale, California). Maximum cellular LDH activity was measured in cell lysates obtained by treatment with Lysis buffer 1X solution. Data from control and treated cells were calculated as percent LDH leakage (100 × LDH activity in medium/maximum LDH activity) and expressed as the mean, using triplicate wells per concentration. The same protocol was performed in parallel without seeding cells for checking possible interaction between NPs and the reagent.

#### Assessment of cell viability

PrestoBlue® (Thermo Fisher, USA) was used for cell viability measurements. This resazurin-based solution was used to quantify the reducing power of living cells as a cell health indicator. After 24 h treatment cells were washed twice with PBS 1X. Fresh media containing PrestoBlue 1X reagent was added to the cells and incubated at 37 °C for 10 min. Fluorescence was detected using excitation and emission pair of 560/590 nm using SpectraMax M5/M5e. Possible interaction of NPs with the reagent was evaluated as mentioned before.

#### Measurement of intracellular reactive oxygen species

The induction of oxidative stress was measured by using both CellRox Green (Invitrogen) and CM-H_2_DCFDA (Invitrogen) in separate experiments. After 24 h of treatment and followed by two washes with 1X PBS, CellRox Green was diluted to 10 μM in media without FBS, added to the cells and incubated at 37 °C for 30 mins. Two washes with 1X PBS were done before measuring fluorescence by SpectraMax M5/M5e, using excitation and emission pair of 485/520 nm. Again, media only and media with NPs were assessed to ensure interference-free measurements. For CM-H_2_DCFDA, after 24 h of cell seeding, the probe was diluted to 10 μM in RPMI media without FBS and added to the cells for 40 mins at 37 °C. After the incubation cells were washed twice with PBS and nanoparticle treatment was applied. Cell imaging was done at 24 and 72 h using InCell analyzer 6000 (GE Healthcare LifeSciences) in epifluorescence mode. Four different fields were acquired for each well. CM-H_2_DCFDA fluorescence (488/510 nm excitation/emission) was acquired at a laser power of 100% and exposure of 400 ms. Images were processed using FIJI software.

### Cellular statistical analysis

Results were expressed as mean ± SD of three independent experiments. Data were analyzed using two-way analysis of variance (ANOVA) with Tukey’s multiple comparison test to determine statistical significance among treatments. In all cases *p* < 0.05 was considered significant.

## Results

### Physicochemical characterization of SiO_2_ NPs

**Fig. 1:**
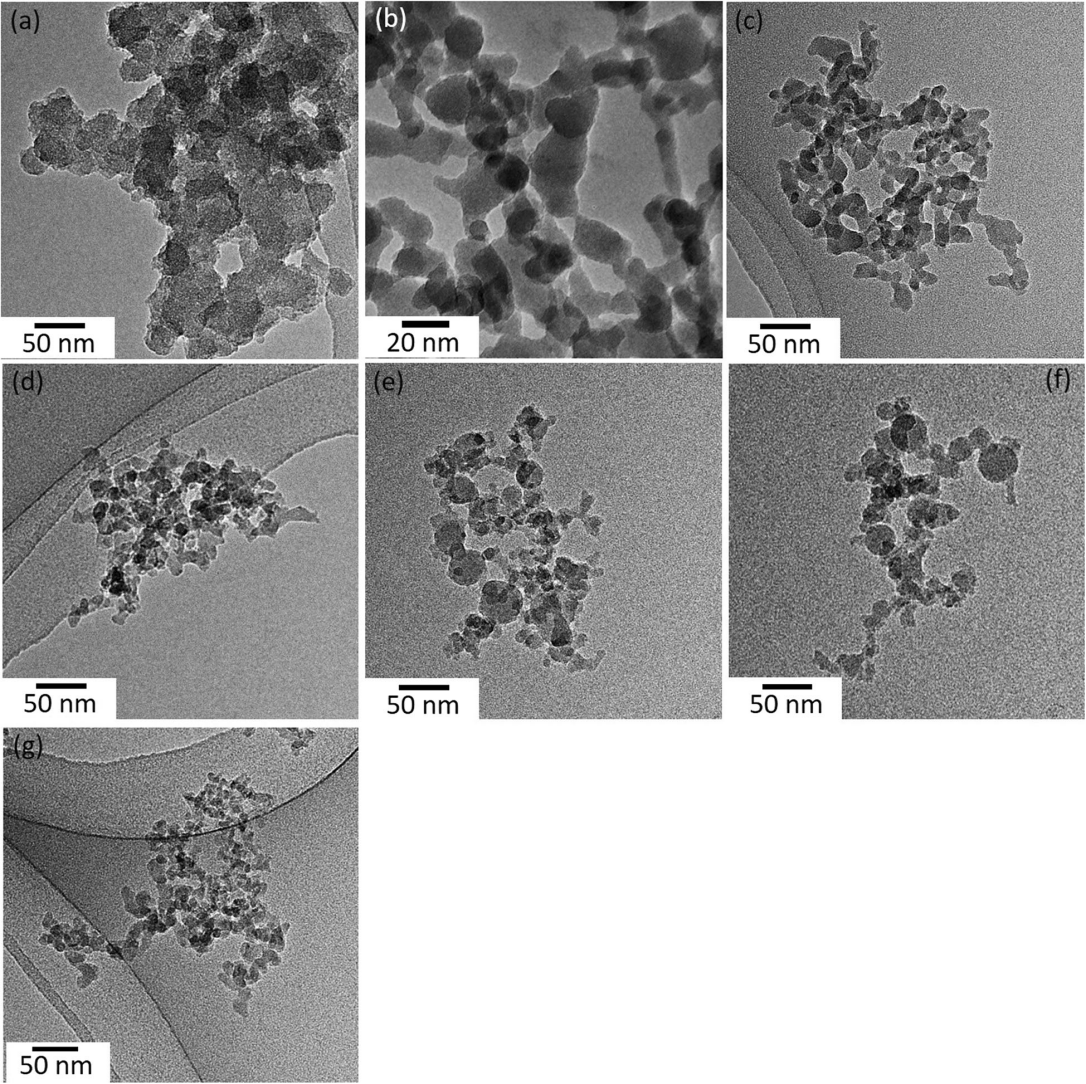
Bright-field TEM images of (**a**) WetChem SiO_2_ and FSP SiO_2_ (**b**) 5/5, (**c**) 3/5, (**d**) 4/5, (**e**) 9/3 and (**f**) 11/3, and (**g**) commercial fumed SiO_2_

Figure 1 depicts the TEM images of SiO_2_ NPs. Imaging analysis indicates that the chain-like agglomerates of SiO_2_ NPs consist of well-defined spherical primary particles. It was observed that the particles of FSP SiO_2_ 9/3 (Fig. 1e), 11/3 (Fig. 1f) and commercial fumed SiO_2_ (Fig. 1g) (produced at higher temperatures, hot-flame synthesis) show limited sinter neck formation, while the particles of FSP SiO_2_ 5/5 (Fig. 1b), 3/5 (Fig. 1c), 4/5 (Fig. 1d) (produced at lower temperatures, cold-flame synthesis) are strongly fused [18]. Further analysis on the surface chemistry, physical characteristics, and chemical purity has been described in detail elsewhere. Previous work has shown that the FSP generated particles are completely amorphous [42].

The SSA values and equivalent primary particle sizes of the samples are outlined in Table 1. For FSP SiO_2_ 3/5, 4/5, 9/3 and 11/3, increasing the feed enthalpy density first increases and then decreases the product SSA (from 186 to 217 m^2^/g and vice versa). It should be mentioned that the study design intentionally created that two pairs of samples exhibit practically equal SSA (FSP SiO_2_ 3/5, 11/3 and FSP SiO_2_ 4/5, 9/3), although the flame conditions are largely different. The BET equivalent diameters ranged from 5 to 15 nm. The density values listed in Table 1 were similar to their bulk counterparts.

**Table 1:** Synthesis, physicochemical parameters, and silanol content of wet chemistry made silica, FSP-made silicas, and commercial fumed silica

Silica type		HDMSO Molarity (M)	x/y	Enthalpy (MJ/kg)	SSA (m^2^/g)	d_BET_ (nm)	Density (g/cc)	Silanol content (mmol OH/g)	SiOx (x)	Si-OH/SiO Ratio
	WetChem SiO_2_	N/A	N/A	N/A	500^a^	4.9	2.4 ± 0.0	5.9 ± 0.0^a^	2.11 ± 0.01	0.65 ± 0.09
Cold Silica	FSP SiO_2_–5/5	0.5	5/5	10.9	152	15.4	2.6 ± 0.1	6.1 ± 0.1	2.01 ± 0.01	0.45 ± 0.06
	FSP SiO_2−_3/5	0.8	3/5	8.9	186^a^	14.7^a^	2.2 ± 0.0	5.2 ± 0.1^a^	2.17 ± 0.01	0.43 ± 0.06
	FSP SiO_2−_4/5	0.8	4/5	10.1	217^a^	12.6^a^	2.2 ± 0.0	4.8 ± 0.1^a^	2.17 ± 0.04	0.35 ± 0.05
Hot Silica	FSP SiO_2−_9/3	0.8	9/3	16.6	217^a^	12.6^a^	2.2 ± 0.0	2.0 ± 0.1^a^	2.06 ± 0.05	0.13 ± 0.02
	FSP SiO_2−_11/3	0.8	11/3	17.8	186^a^	14.7^a^	2.2 ± 0.0	1.6 ± 0.1^a^	2.11 ± 0.05	0.11 ± 0.01
	Comm. fumed SiO_2_	N/A	N/A	N/A	206^a^	9.0^a^	3.2 ± 0.1	0.7 ± 0.0^a^	2.02 ± 0.04	0.07 ± 0.01

^a^The values are from reference [18]

*SSA* Specific surface area, *d*_*BET*_ Diameter determined by Brunauer-Emmett-Teller nitrogen adsorption, *FSP* Flame spray pyrolysis; The nomenclature used in this table for Silica NPs indicates the method of synthesis (FSP), the molarity, and the precursor/dispersion flows ratio x/y

Table 1 also includes the total silanol content of the different SiO_2_ NPs and the enthalpy of the flame used to synthesize the NPs. The lowest silanol content belongs to the commercial fumed silica sample (0.7 mmol OH/g). The highest two values belonged to the FSP SiO_2_ 5/5 (6.1 mmol OH/g) followed by the WetChem SiO_2_ NPs (5.9 mmol OH/g). An example of the TGA graph and the related analysis for the FSP SiO_2_ 5/5 is described in Additional file 1: Figure S1.

The XPS revealed that the particles are highly stoichiometric without significant variation among the various silica particles (Table 1) with very low residual carbon indicating complete combustion of the precursor mix (< 2%). Further, the oxygen peak was used to calculate the Si-OH/O-Si-O bond ratio localized on the surface (Table 1). The Si-OH/Si-O bond ratio as a function of the enthalpy of the flame used to synthesize the NPs shows a relationship that is similar to the one calculated from the TGA measurements (Additional file 1: Figure S2a).

### Suspension preparation, colloidal characterization and dosimetry analysis of SiO_2_ NPs

The DSE_cr_ used to disperse SiO_2_ NPs along with their colloidal properties are summarized in Additional file 1: Table S1 and Additional file 1: Figure S3. The SiO_2_ NPs agglomerates were stable over 24 h in the physiological medium (RPMI 10% FBS), which was confirmed by serial DLS measurements (Additional file 1: Table S1). The mean effective density values of SiO_2_ NPs agglomerates range between 1.12 and 1.31 g/cm^3^, lowest value belonging to the WetChem SiO_2_.

The normalized delivered-to-cell mass concentrations and fractions deposited as a function of the exposure time are plotted in Additional file 1: Figure S4. Based on these results, it is inferred that SiO_2_ NPs samples were well dispersed, and the agglomerates settle gradually to reach the maximum concentration at the bottom of the well. All FSP SiO_2_ NPs display extremely slow settling, with no more than 1% of the administered dose deposited on the cells after 24 h of exposure. More importantly, however, the fraction deposited varies greatly between all SiO_2_ NPs, which points to the importance of including dosimetric analysis in such a study.

### Acellular ROS production

Table 2 summarizes the rates of production of short-lived ROS and H_2_O_2_, expressed as slopes of the dose-response curves (pmol H_2_O_2_ eq./μg material) for the seven types of SiO_2_ NPs over the dose range of 10–100 μg/ml. When the dose is expressed as mass concentration (μg/ml), the dose-response curve is linear over the tested dose range (Additional file 1: Table S2). The short-lived ROS produced by different SiO_2_ NPs ranged from 4.8 to 20.3 pmol H_2_O_2_ eq./μg silica. The production of H_2_O_2_ varied from 22.9 to 43.0 pmol/μg SiO_2_ NPs. The total ROS (sum of short-lived ROS and H_2_O_2_) ranged from 33.5 to 67.2 pmol/μg SiO_2_ NPs. Short-lived ROS accounted for 13.0 to 47% of total ROS, averaging at 30.6% for all materials. For short-lived ROS, the highest value was measured for FSP 11/3 (21 pmol/μg), whereas the lowest rate was for WetChem (4.3 pmol/μg). For H_2_O_2_, the highest value belonged to FSP 9/3 SiO_2_ (50.2 pmol/μg), whereas the lowest value to FSP 5/5 SiO_2_ NPs (22.9 pmol/μg). With regards to the total ROS production, the highest rate was for FSP 9/3, whereas the lowest rate was for FSP 4/5. The group of “Hot SiO_2_ NPs” (FSP SiO_2_ 9/3, 11/3 and commercial fumed SiO_2_) produced higher short-lived ROS, H_2_O_2_, and total ROS than the group of “cold SiO_2_ NPs” (FSP SiO_2_ 5/5, 3/5, 4/5, and Wetchem SiO_2_).

**Table 2:** Slopes of dose-response curves for short-lived ROS and H_2_O_2_ generated by seven silica NPs over the concentration range of 10 to 100 μg/ml

Slope	Silica nanoparticle type						
	WetChem	FSP 5/5	FSP 3/5	FSP 4/5	FSP 9/3	FSP 11/3	Commercial
Short-lived ROS (pmol H_2_O_2_ eq./μg silica)	4.8	20.3	11.8	8.3	17	21	19.6
*Ratio relative to WetChem (short-lived)*	*1.0*	*4.2*	*2.5*	*1.7*	*3.5*	*4.4*	4.1
H_2_O_2_ (pmol H_2_O_2_ eq./μg silica)	32	22.9	26.4	25.3	50.2	41.7	43
Ratio relative to WetChem (H_2_O_2_)	1.00	0.72	0.83	0.79	1.57	1.3	1.34
Total ROS(pmol H_2_O_2_ eq./μg silica)	36.8	43.2	38.1	33.5	67.2	62.7	62.6
Ratio relative to WetChem (total ROS)	1.00	1.2	1.00	0.91	1.83	1.7	1.70
Short-lived, % of total ROS	13.0	47.0	31.0	24.8	33.9	33.5	31.3

**Fig. 2:**
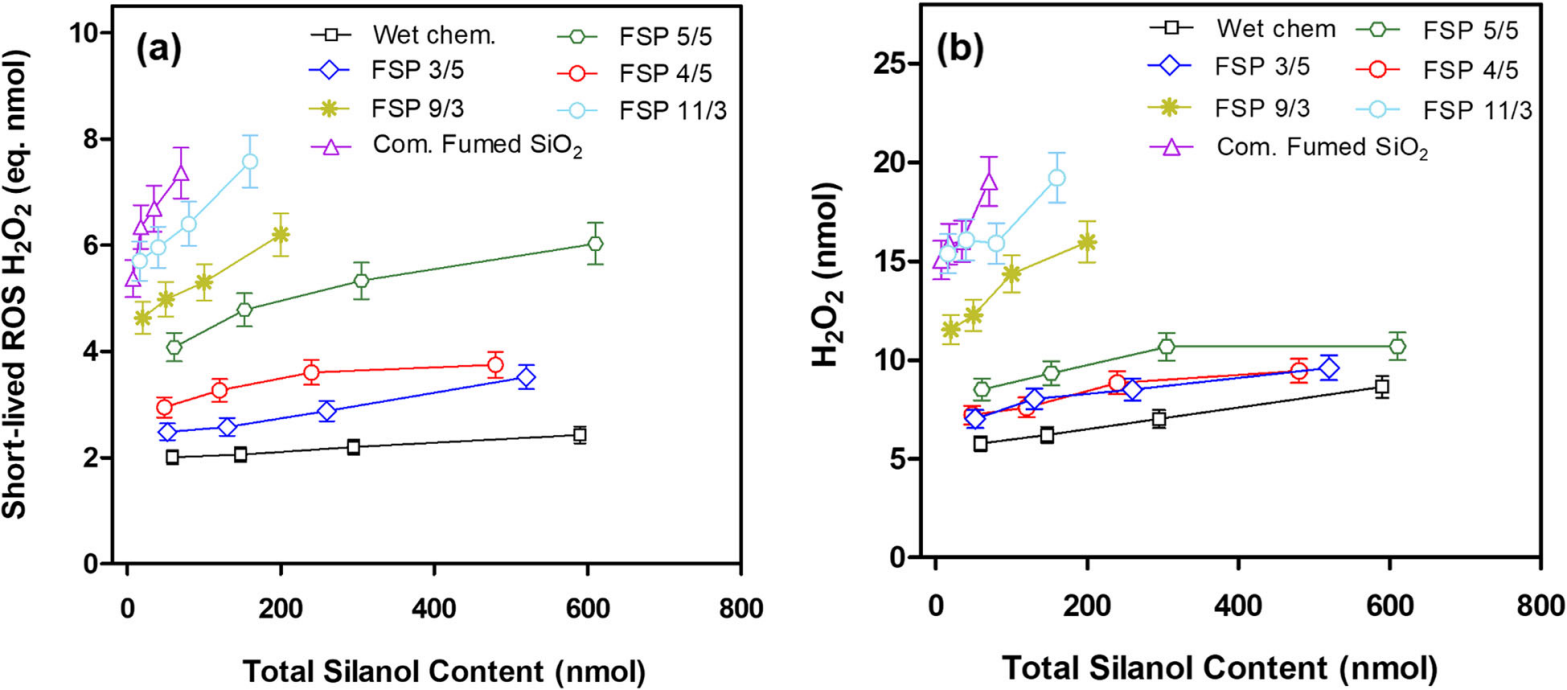
Amount of the short-lived ROS (**a**) and H_2_O_2_ (**b**) generated from seven silicas as a function of the total silanol content over the 10 to 100 μg NP dose range

In order to study the influence of silanol group content on acellular ROS production, the dose of SiO_2_ NPs was calculated with both SSA and total silanol groups (see Table 1) assuming that the ratio of surface to bulk silanol groups is approximately the same [18]. The data are presented in Fig. 2 (short-lived ROS and H_2_O_2_). It should be noted that within each material, the total silanol content on the x-axis is proportional to SSA. In general, the content of short-lived ROS and H_2_O_2_ production within each material was proportional to the number of silanol groups. As for short-lived ROS, when normalized to silanol content, WetChem SiO_2_ exhibited the lowest rate of short-lived ROS production compared to all other silicas, whereas the commercial fumed SiO_2_ NPs sample exhibited the highest rate of short-lived ROS formation (Fig. 2a). The ratio of short-lived ROS production between commercial fumed and Wetchem SiO_2_ was 35-fold. FSP SiO_2_ 5/5 produced higher ROS than FSP SiO_2_ 3/5 and FSP SiO_2_ 4/5, but lower than FSP SiO_2_ 9/3 and 11/3 (“hot SiO_2_ NPs”). As for H_2_O_2_ production (Fig. 2b), although the rank order of silicas in H_2_O_2_ formation is similar to the short-lived ROS, the two groups are markedly separated from each other. Conversion of fast-reacting ROS, such as hydroxyl radicals, into H_2_O_2_ and/or its direct synthesis on the silica surfaces, appears to depend on the surface properties of SiO_2_ NPs. Although the general trend is that silicas containing lower silanol content produce more acellular ROS (short-lived ROS and H_2_O_2_) (Fig. 2a,b), it should be noted that at any fixed value of silanol content (e.g. 75 nmol), each material produces different amounts of ROS (Additional file 1: Figure S5a and b).

### In vitro cellular toxicity

#### Silanol content plays a role in cell membrane integrity and viability

**Fig. 3:**
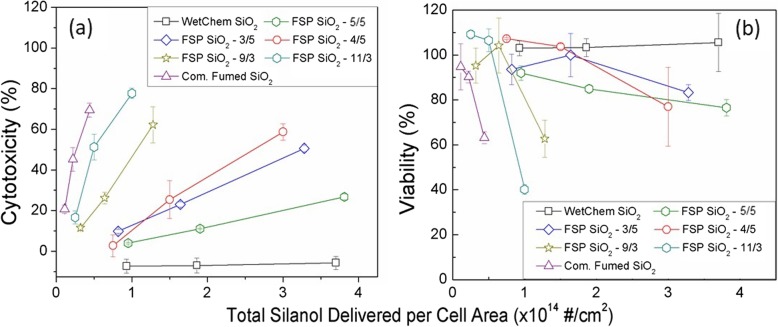
**a** Cytotoxicity, compared to the negative control (untreated cells), as measured by the LDH Cytotoxicity Assay Kit; and (**b**) Viability, compared to the negative control, (PrestoBlue assay) measured in the RAW264.7 macrophages. The x-axis represents the total delivered-to-cells silanol group, per cell area, adjusted for dosimetry, for the three administered doses. Data represent the average and the standard error of three independent experiments performed in triplicate

To study the effect of silanol content in cell response, RAW 264.7 and SAEC were treated with the panel of SiO_2_ NPs covering a wide range of silanol content for 24 h. SAEC were not sensitive enough to yield any significant change in LDH release indicating that cell membrane integrity was not compromised (Additional file 1: Figure S6a) and no measurable cell necrosis occurred. However, in RAW 264.7 macrophage cells, LDH levels increased inversely proportional to the amount of silanol content delivered to the cells for all tested NPs, except for WetChem SiO_2_ for which no LDH activity was detected (Fig. 3a). No interaction of NPs with the reagent was observed in any of the cases studied. The patterns in Fig. 3a resemble closely those in Fig. 2a, especially for the “hot SiO2 NPs”. This similarity is explained by the linear relation that can be also be established between the acellular ROS and the cytotoxicity (Additional file 1: Figure S8). The steep dose-response slopes observed in Fig. 3a provide evidence of significant membranolysis by “hot SiO_2_ NPs” over the delivered dose range (from 15 to 20% of cells at the lowest delivered dose to 70–80% at the high dose). “Cold SiO_2_ NPs”, which contained higher silanol content, caused significantly less cell damage via necrosis, especially at the same total silanol load. The slopes of dose-response for the “cold SiO_2_ NPs” are notably less steep than for the “hot SiO_2_ NPs”. Furthermore, each material exhibited its own unique dose-response slope, regardless of the synthesis group (Fig. 3a and Additional file 1: Figure S5c). This is compelling evidence that the silanol content is not the only factor affecting LDH release and the membranolytic/necrotic properties of amorphous silica.

Similar to cell membrane integrity, cell viability results show no toxicity for all types of silica NPs in the SAEC cell line (Additional file 1: Figure S6b). A significant delivered dose-dependent decrease in cell viability was shown for RAW 264.7 cells (Fig. 3b). Generally, the same pattern of toxicity and dose-response was observed for cell viability (PrestoBlue assay) as in the LDH assay: the “hot SiO_2_ NPs” with lower silanol content exhibited higher toxicity. However, when normalized on the basis of the total silanol content delivered per cell area, surface properties other than silanol content are again appearing to influence cellular toxicity (Additional file 1: Figure S5d). The two commercial samples represented the least (WetChem Silica) and the most toxic NPs (commercial fumed silica), respectively.

Another endpoint that validates the cell membrane integrity and cell viability results are cellular proliferation observed by microscopy (Fig. 5). As presented in Fig. 5b, after 72 h of treatment, “cold SiO_2_ NPs” (5/5, 3/5, 4/5) showed unhindered cell growth similar to untreated cells (negative control) (Fig. 5a). However, in cells treated with “hot SiO_2_ NPs”, cell growth was inhibited compared to the negative control, and a change in cell morphology from round to elongated was also observed (Fig. 5b). This is suggestive of additional harmful effects on normal cell function induced by “hot SiO_2_ NPs”, which requires further investigation.

#### Intracellular ROS

**Fig. 4:**
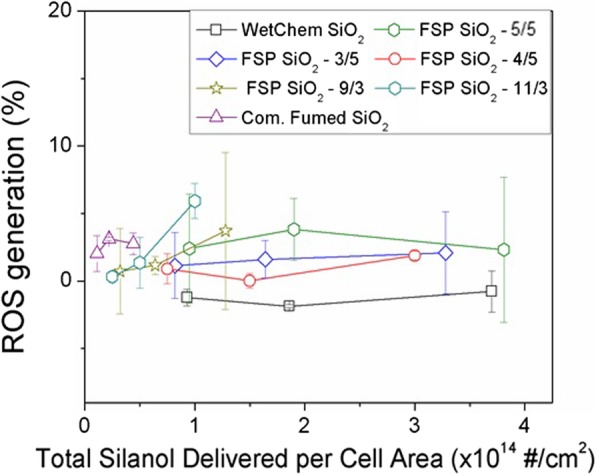
Intracellular ROS generation, compared to the negative control, in RAW264.7 cells after 24-h treatment measured with the CellROX Green assay. The silica NP dose is reported as the total silanol delivered per cell area over the range of three administered doses. Data represent the average and the standard error of three independent experiments performed in triplicate

**Fig. 5:**
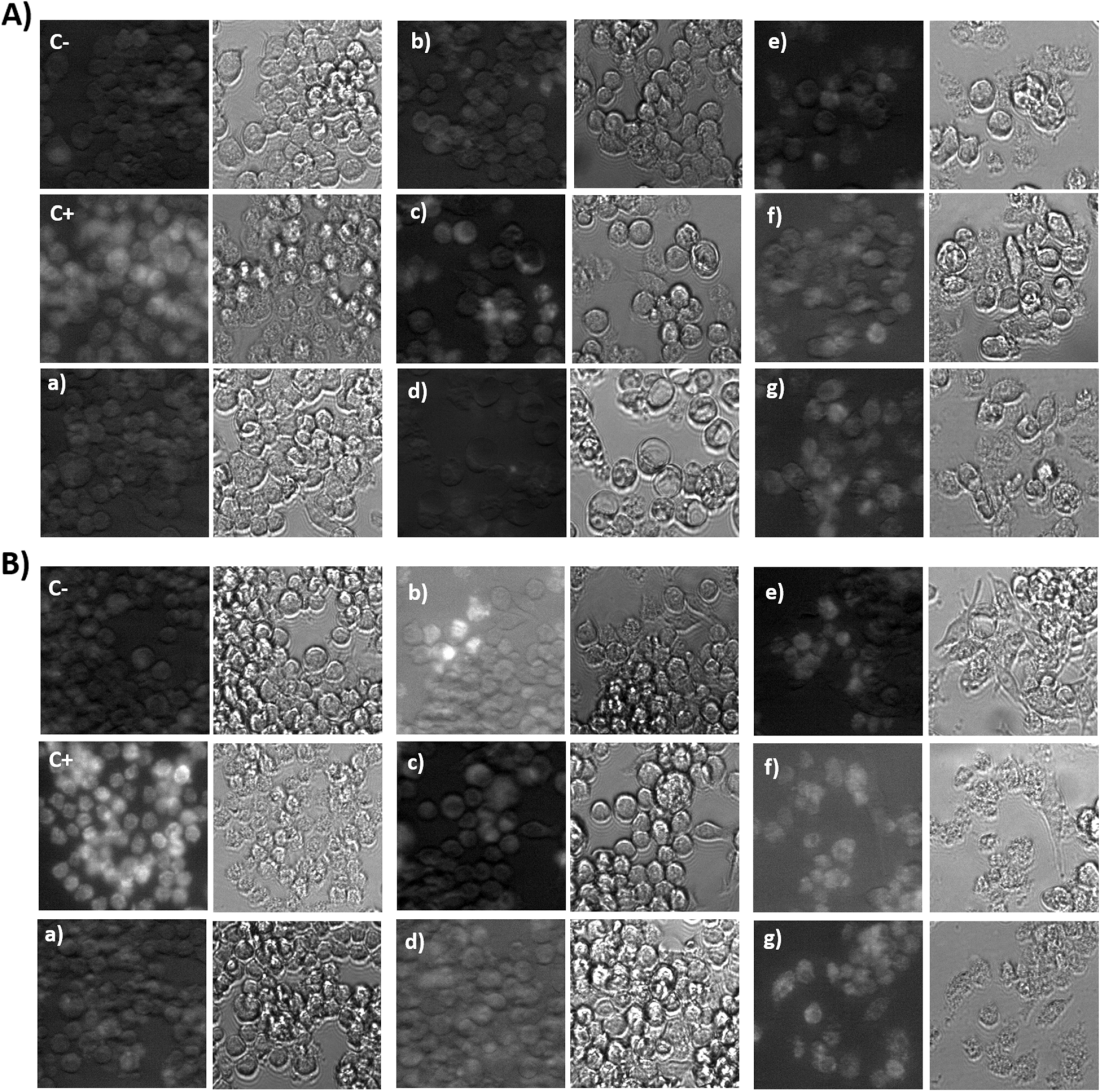
Confocal images of intracellular ROS generation as a measure of CM-H_2_DCFDA fluorescence in RAW264.7 cells treated with the highest delivered concentration (0.104 μg/cm^2^) after 24 h (A) and 72 h (B). Lowercase letters correspond to the different SiO_2_ NPs: (**a**) WetChem SiO_2_ and FSP SiO_2_ (**b**) 5/5, (**c**) 3/5, (**d**) 4/5, (**e**) 9/3 and (**f**) 11/3, and (**g**) commercial fumed SiO_2_. Controls: (C-) negative control and (C+) positive control treated with H_2_O_2._ . Two images were obtained corresponding to green fluorescence channel (left) and transmitted light image (right)

Intracellular ROS production was determined after 24 h of incubation with the panel of SiO_2_ NPs. No statistically significant increase in ROS production, as measured with the CellRox assay, was seen in RAW264.7 (Fig. 4) and SAEC (Additional file 1: Figure S7) cell lines. Intracellular ROS production was further investigated by confocal microscopy and a slight increase in fluorescence, suggestive of ROS generation, was seen for some silicas at 24 h. For the highest dose, all materials showed low fluorescence signal, except for the commercial fumed SiO_2,_ and FSP SiO_2_ 11/3 SiO_2,_ which produced a higher signal. After 72 h of exposure (Fig. 5b), the same trend was confirmed indicating that “hot SiO_2_ NPs” show the highest intracellular ROS production although high toxicity was observed.

## Discussion

Inhalation toxicology of respirable crystalline silica has been reported extensively in the published literature. Crystallinity and bio-persistence of crystalline silica play an important role in the development of various lung diseases, such as silicosis, lung cancer, or emphysema [55, 56]. Amorphous silica, contrary to crystalline silica, is seen as much less toxic. As we show in this work, surface physicochemical properties of amorphous silica can change considerably, depending on the production conditions, leading to materials with a range of toxicities [20, 57]. It has been postulated that toxicity of especially amorphous SiO_2_ is related to the number of the silanol groups on their surfaces, a property that depends largely on the combustion enthalpy/synthesis method [56, 58]. Specifically, fumed SiO_2_ that is produced under hot temperature conditions, and contain low silanol content, has been shown in vitro to induce cytotoxicity, apoptosis, genotoxicity, oxidative stress and pro-inflammatory mediators [21–24].

In this work, a series of well-characterized synthesized FSP amorphous SiO_2_ [18, 42] was used to investigate the role of silanol content in ROS production and cellular toxicity. In addition to the FSP SiO_2_ NPs, two commercially available NPs - a fumed silica and silica synthesized via wet chemistry methods - were used as positive and negative controls, respectively. For the FSP silicas, the total silanol content (surface and internal silanols), as quantified by TGA, decreased with increasing combustion enthalpy [18, 48, 59], which in turn is a function of the operation parameters of the FSP (Table 1). This was also confirmed with XPS that showed the variation of the Si-OH/O-Si-O ratio varying as a function of the combustion enthalpy (Additional file 1: Figure S2a). XPS is a surface-sensitive method that interrogates the first 1–2 nm of the ENM surface and therefore the Si-O-H signal measured is primarily due to the surface-bound silanol. Although the exact quantification is not possible through XPS, it can be concluded that the “surface” silanol groups on the first 1–2 nm are proportional to the total silanol groups (Additional file 1: Figure S2b). The WetChem silica and the commercially available fumed silica have respectively the highest and lowest silanol content. The FSP SiO_2_ 5/5 is a material with very high silanol content and is part of the HSPH-NIEHS Reference ENMs Repository and used as a reference ENM for toxicological studies.

It should be highlighted that the FSP SiO_2_ 4/5 and FSP SiO_2_ 9/3 NPs were designed to have equal SSA values (217 m^2^/g) and primary particle diameters, but different total silanol content (4.8 vs. 2.0 mmol OH/g respectively). Similarly, the other pair of FSP, SiO_2_ 3/5 and FSP SiO_2_ 11/3, with the same SSA (186 m^2^/g), have different silanol content (5.2 and 1.6 mmol OH/g, respectively). Since the surface area is different, normalizing silanol content to the surface area enables a direct comparison of the different SiO_2_ NPs. Therefore, the toxicological studies were performed on the bases of surface area delivered to the cells and not on mass delivered to the cells.

For the toxicological assessment, a parameter that is often overlooked is the cellular dosimetry. In particular, for nanoparticles, the dose delivered to the cell and the administered dose can vary up to two orders of magnitude [39, 40]. The delivered dose to cells is a critical factor since dose metrics are particularly important for in vitro cellular toxicity assessment [51]. More importantly, direct contact of the NPs with cellular membranes is the first step in the sequence of cellular events leading to NP uptake and cytotoxicity, and therefore estimating the correct fraction of particles deposited on the cells as a function of exposure time is of paramount importance for in vitro dose-response assessments [41, 51, 60]. In contrast to previous studies which assume that all administered nanoparticles settle on top of the cells, our results confirm that the delivered doses for amorphous silicas are substantially smaller than the administered dose, by a factor of ~ 100. This is significant in light of the fact that no more than 1% of the administered dose reaches the cells. More importantly, the delivered to cell dose differs among the various SiO_2_ NPs used in this study by a factor of 2, a difference that we took into consideration in dose estimates in this study. The lack of dosimetry considerations in previous studies makes it impossible to compare reliably the dose-response slopes across various endpoints for multiple NPs, solely based on administered dose. The net effect of ignoring dosimetry considerations when comparing multiple NPs is lack of power to detect small differences between NPs.

Surface properties, such as surface activity and oxidative stress potential of NPs are important predictors of biological activity and toxicity in structure-activity relationships (SAR) [61]. Characterizing the surface chemistry of amorphous silica NPs and, more specifically, quantifying their silanol content and exploring links with the bioactivity of these NPs was the main objective of this study. It is worth pointing that, as shown previously by the authors using Raman and diffuse reflectance infrared Fourier spectroscopy analysis, there is a marked relationship between the temperature of FSP SiO_2_ synthesis and the distribution of isolated vs. hydrogen bonded silanol groups. Thereby, higher synthesis temperatures lead to lower total silanol content, which corresponds to a higher frequency of isolated silanol groups. These specific groups could contribute to stronger hydrogen bonds and/or higher electrostatic interactions between isolated silanols and cell membranes [32]. On the other hand, WetChem SiO_2_ synthesized at very low temperatures showed lower proportion of isolated groups [18], which accordingly did not show any damage to the cell membrane or any effect on viability. This is consistent with other studies showing the implication of isolated silanol groups as a mechanism of cell toxicity [20, 62, 63].

Understanding how SiO_2_ nanoparticles by themselves generate ROS in acellular environments gives insight into the related potential toxicity and could provide insights into the toxicological mechanism. In general, our results seem to be similar to the in vitro cytotoxicity experiments. SiO_2_ NPs with a higher silanol content showed lower acellular ROS production, whilst SiO_2_ NPs particles containing low silanol content (FSP 9/3, 11/3 and commercial fumed SiO_2_) resulted in higher ROS production. In agreement with these findings, Zhang et al. found fumed silica more capable of generating hydroxyl radicals than SiO_2_ NPs synthesized through low-temperature approaches [20]. An important aspect presented in this study is quantitation of short-lived ROS and H_2_O_2_. H_2_O_2_ triggers different signaling pathways than short-lived ROS. While short-lived ROS are unstable radicals that could directly oxidize cellular components, H_2_O_2_ is relatively stable and is a key factor modulating many cellular functions. Thus, H_2_O_2_ activates different cellular pathways by different mechanisms such as gene activation or production of pro-oxidants involved in apoptosis among many others [64].

Intracellular ROS production as a consequence of SiO_2_ NPs exposure has been described in several studies [15, 65, 66],. To correlate acellular to cellular results, intracellular ROS was investigated. However, although acellular ROS production was quantified in all NPs in a dose-dependent manner, ROS was difficult to observe in intracellular environments. This is could be the result of limited sensitivity of the optical assays employed for intracellular ROS measurements, which includes the CellROX assay and the confocal imaging of fluorophores. In this case, using confocal microscopy RAW264.7 showed a slight increase in ROS with the highest values corresponding to commercial fumed SiO_2_ and FSP 11/3 (Fig. 5). Regarding CellROX results, intracellular ROS production was not high enough to be detected in any cell line. This could be due to a lack of assay sensitivity, and it follows that in future studies of this kind, monitoring the expression of antioxidant genes, such as HO1, GPX1, and SOD, maybe a more sensitive approach. Alternatively, this discrepancy may also suggest a different mechanism of toxicity whereby extracellular ROS disrupt the cellular membranes once NPs are being taken up by macrophages, as shown in the LDH results (see Fig. 3), leading to cell necrosis. In this context, Murugados et al. reported that most studies on the toxicity of fumed SiO_2_ NPs show a non-ROS related mechanism of intracellular damage [56].

Our data show that SiO_2_ NPs demonstrated no significant toxicity for the SAEC cell line although direct contact was achieved, as was shown by the DG model. This highlight the importance of the cell type choice when assessing SiO_2_ NPs toxicity. This cell-dependent effect has been described extensively in the published literature and could reflect distinct physiological functions of cell types including their ability to phagocytose NPs [67]. It is well known that macrophages, such as RAW264.7 cells, efficiently take up particles through endocytosis and phagocytosis [68, 69]. This uptake of SiO_2_ NPs and their potential to damage endolysosomal vesicles in which they reside following particle uptake can likely modulate the cytotoxic response [70]. As such, these cell-dependent physiological functions may explain non-toxic response found in SAEC epithelial cells.

Although we have demonstrated how silanol content is an important factor modulating amorphous fumed SiO_2_ NPs toxicity, the data document clearly that some other surface activity features – reflected remarkably well in the acellular ROS assays as different surface activity indexes, contribute to these silicas toxicity (Additional file 1: Figure S5). However, due to the importance of surface chemistry in cell response, controlling silanol content and, in turn, the content of isolated silanol groups is a good choice for a safe-by-design approach. In the same direction with the intention of decreasing toxicity, Sun et al. modified fumed SiO_2_ NPs surface chemistry by calcination and doping [19]. Although both methods led to a significant decrease in the inflammatory effects in vivo and in vitro, some potential pitfalls could be identified. Calcination is indeed sensitive to moisture and adsorption of contaminants on the NP surface, which may influence downstream toxicity responses. In addition, this thermal treatment does not yield stable surface properties, reverse reactions that depend on the surrounding microenvironments, may reactivate NP surfaces potentially causing a recovery in toxicity [19]. On the other hand, although doping with elements does not suffer from this drawback, it could change the silica functionality when used in commercially-enabled products, which still needs to be verified.

Finally, it is worth discussing hemolysis, the rupture of red blood cells membrane due to interactions with nanoparticles, which is a well-known/studied in the literature effect of SiO_2_ NPs. Hemolysis is believed to be induced to a great extent by silanol groups [32]. In this current study, we observed that the cell membrane damage in RAW 264.7 macrophages (LDH assay), was proportional to the total silanol content delivered to cells. As Fig. 3a shows, SiO_2_ NPs with very similar SSA and colloidal properties, but different silanol content, induced different LDH responses. Specifically, “hot SiO_2_ NPs” with lower silanol content were more cytotoxic (judged by LDH release and cell viability) than the “cold SiO_2_ NPs” with higher silanol content. However, when comparing the slopes of cytotoxicity/viability vs. delivered silanol dose, the effect produced was distinctive for each material at the same silanol dose delivered to the cell. Therefore, although particles with lower silanol content show higher toxicity compared to particles with higher silanol content, other factors seem to be contributing to the cytotoxic effects of SiO_2_ NPs. Thus, apart from the synthesis process, the silanol content and the siloxane group density, other factors already mentioned in literature, such as morphology, roughness or porosity could be also playing a role in silica-induced toxicity [20, 31, 32].

Taken altogether, this study shows the importance of silanol content in cell toxicity of RAW264.7 cell line. A safer-by-design synthesis approach can be derived based on the results of this study. Indeed, by controlling the FSP combustion enthalpy during NP synthesis, the silanol content and other surface properties can also be modulated, resulting in milder cellular effects.

## Conclusions

In this work, silanol content is shown to be one of the major factors affecting amorphous silica toxicity. Flame synthesis, which is highly used in the industry as a synthesis process, can be finely tuned to modulate the specific amount of silanol groups on SiO_2_ NPs by controlling combustion enthalpy and flame conditions. It was clearly demonstrated in this study that a lower toxic dose-response is observed with increasing total silanol content, probably related to a decrease in isolated silanol groups or an increase in the siloxane bridges. Apart from silanol content, other factors such as cell line functionality and silanol organization on the surface may also affect amorphous SiO_2_ NP toxicity. Nevertheless, due to the clear impact of silanol content in cell toxicity, a safer by design synthesis approach for FSP SiO_2_ NPs is recommended, based on utilizing low enthalpies of combustion.

## Supplementary information

**Additional file 1: Figure S1.** TGA temperature-time profile (right ordinate, dashed line), corresponding sample mass (left ordinate) of as-produced (solid lines) and the mass loss normalized to the mass at the end of Step 1. **Figure S2.** XPS analysis. (a) Si-OH/O-Si-O ratio and total silanol content varying as a function of the combustion enthalpy. (b) Si-OH/O-Si-O ratio as a function of the total silanol. **Figure S3.** Determining the critical delivered sonication energy of SiO_2_ NPs. (a) Mean hydrodynamic diameter and (b) polydispersity index as a function of dispersion sonication energy of Wetchem SiO_2_ NPs, FSP made SiO_2_ NPs and commercial fumed SiO_2_ NPs in DI H_2_O. **Figure S4.** Fate and transport modeling results for SiO_2_ NPs. (a) Delivered-to-cell concentration normalized to the administered dose and (b) delivered-to-cell fraction deposited of wet chemistry made silica, FSP made SiO_2_ NPs and commercial fumed SiO_2_ NPs in RPMI + 10% (vol/vol) FBS. Solid lines are the fitting curves obtained using eq. 1 and 2. **Figure S5.** Importance of other modulators in silica NPs effect analyzing RAW264.7 cells. (a, b) short-lived ROS and H_2_O_2_ produced by the different SiO_2_ NPs at a fixed value of silanol content of 150 nmol. (c) Cytotoxicity of different SiO_2_ NPs at a fixed value of delivered silanol per cell area of 1 × 10^14^ #/cm^2^. (d) Viability of different SiO_2_ NPs at a fixed value of delivered silanol per cell area of 1.5 × 10^14^ #/cm^2^. **Figure S6.** Cytotoxicity (a) and Viability (PrestoBlue assay) (b) measured in SAEC cells. The data represented as function of total silanol delivered per cell area for the three delivered doses used. Data represent an average of three independent experiments performed in triplicate. **Figure S7.** ROS generation as a measure of oxidative damage (CellROX Green assay) in SAEC cells. After 24-h treatment, ROS generation was measured and data represented as function of total silanol delivered per cell area for the three delivered doses used. Data represent an average of three independent experiments performed in triplicate. **Figure S8.** Cytotoxicity measured in RAW264.7 cells. The data is represented as function of short life ROS-H_2_O_2_ eq. nmol. Data represent an average of three independent experiments performed in triplicate. **Table S1.** Mean values of the parameters obtained for suspension preparation and colloidal characterization of wet chemistry made silica, FSP made silicas and commercial fumed silica in H_2_O and RPMI + 10% (vol/vol) FBS. **Table S2.** The short-lived ROS and H_2_O_2_ generated from seven types of silica over the 10–100 μg/mL range. Values have been corrected for sonication and background oxidation of Trolox.

## Data Availability

Not applicable.
