# Supplementary material for: Safer-by-design flame-sprayed silicon dioxide nanoparticles: the role of silanol content on ROS generation, surface activity and cytotoxicity
Source: Part Fibre Toxicol. 2019 Oct 29;16:40. doi: 10.1186/s12989-019-0325-1 (PMC6819463; doi:10.1186/s12989-019-0325-1)
Supplement: Supplementary file 1 — Additional file 1: Figure S1. TGA temperature-time profile (right ordinate, dashed line), corresponding sample mass (left ordinate) of as-produced (solid lines) and the mass loss normalized to the mass at the end of Step 1. Figure S2. XPS analysis. (a) Si-OH/O-Si-O ratio and total silanol content varying as a function of the combustion enthalpy. (b) Si-OH/O-Si-O ratio as a function of the total silanol. Figure S3. Determining the critical delivered sonication energy of SiO2 NPs. (a) Mean hydrodynamic diameter and (b) polydispersity index as a function of dispersion sonication energy of Wetchem SiO2 NPs, FSP made SiO2 NPs and commercial fumed SiO2 NPs in DI H2O. Figure S4. Fate and transport modeling results for SiO2 NPs. (a) Delivered-to-cell concentration normalized to the administered dose and (b) delivered-to-cell fraction deposited of wet chemistry made silica, FSP made SiO2 NPs and commercial fumed SiO2 NPs in RPMI + 10% (vol/vol) FBS. Solid lines are the fitting curves obtained using eq. 1 and 2. Figure S5. Importance of other modulators in silica NPs effect analyzing RAW264.7 cells. (a, b) short-lived ROS and H2O2 produced by the different SiO2 NPs at a fixed value of silanol content of 150 nmol. (c) Cytotoxicity of different SiO2 NPs at a fixed value of delivered silanol per cell area of 1 × 10^14^ #/cm2. (d) Viability of different SiO2 NPs at a fixed value of delivered silanol per cell area of 1.5 × 10^14^ #/cm2. Figure S6. Cytotoxicity (a) and Viability (PrestoBlue assay) (b) measured in SAEC cells. The data represented as function of total silanol delivered per cell area for the three delivered doses used. Data represent an average of three independent experiments performed in triplicate. Figure S7. ROS generation as a measure of oxidative damage (CellROX Green assay) in SAEC cells. After 24-h treatment, ROS generation was measured and data represented as function of total silanol delivered per cell area for the three delivered doses used. Data represent an a [file 12989_2019_325_MOESM1_ESM.docx]

**Safer-by-design flame-sprayed silicon dioxide nanoparticles: The role of silanol content on ROS generation, surface activity and cytotoxicity**

Laura Rubio^a+^, Pyrgiotakis G^a+^ , Juan Beltran-Huarac^a^, Yipei Zhang^b^, Gaurav N. Joshi^c^, Glen DeLoid^a^, Anastasia Spyrogianni^d^, Kristopher A. Sarosiek^c^, Dhimiter Bello^a,b^, and Philip Demokritou^a,^*

^a^ Center for Nanotechnology and Nanotoxicology, HSPH-NIEHS Nanosafety Center, Department of Environmental Health, Harvard T. H. Chan School of Public School, Harvard University, 665 Huntington Boston, MA 02115, USA.

^b^ Department of Biomedical and Nutritional Sciences, Zuckerberg College of Health Sciences, University of Massachusetts Lowell, MA 01854, USA.

^c^ John B. Little Center for Radiation Sciences, Department of Environmental Health, Harvard T.H. Chan School of Public Health, Boston, MA 02115, USA

^d^ Particle Technology Laboratory, Institute of Process Engineering, Department of Mechanical and Process Engineering, ETH Zurich, Sonneggstrasse 3, CH-8092, Zurich, Switzerland

*Corresponding author. E-mail: [pdemokri@hsph.harvard.edu](mailto:pdemokri@hsph.harvard.edu)

^+^Equally contributing authors

**Keywords:** Silanol groups, amorphous silica, chemistry surface, flame spray pyrolysis, toxicity


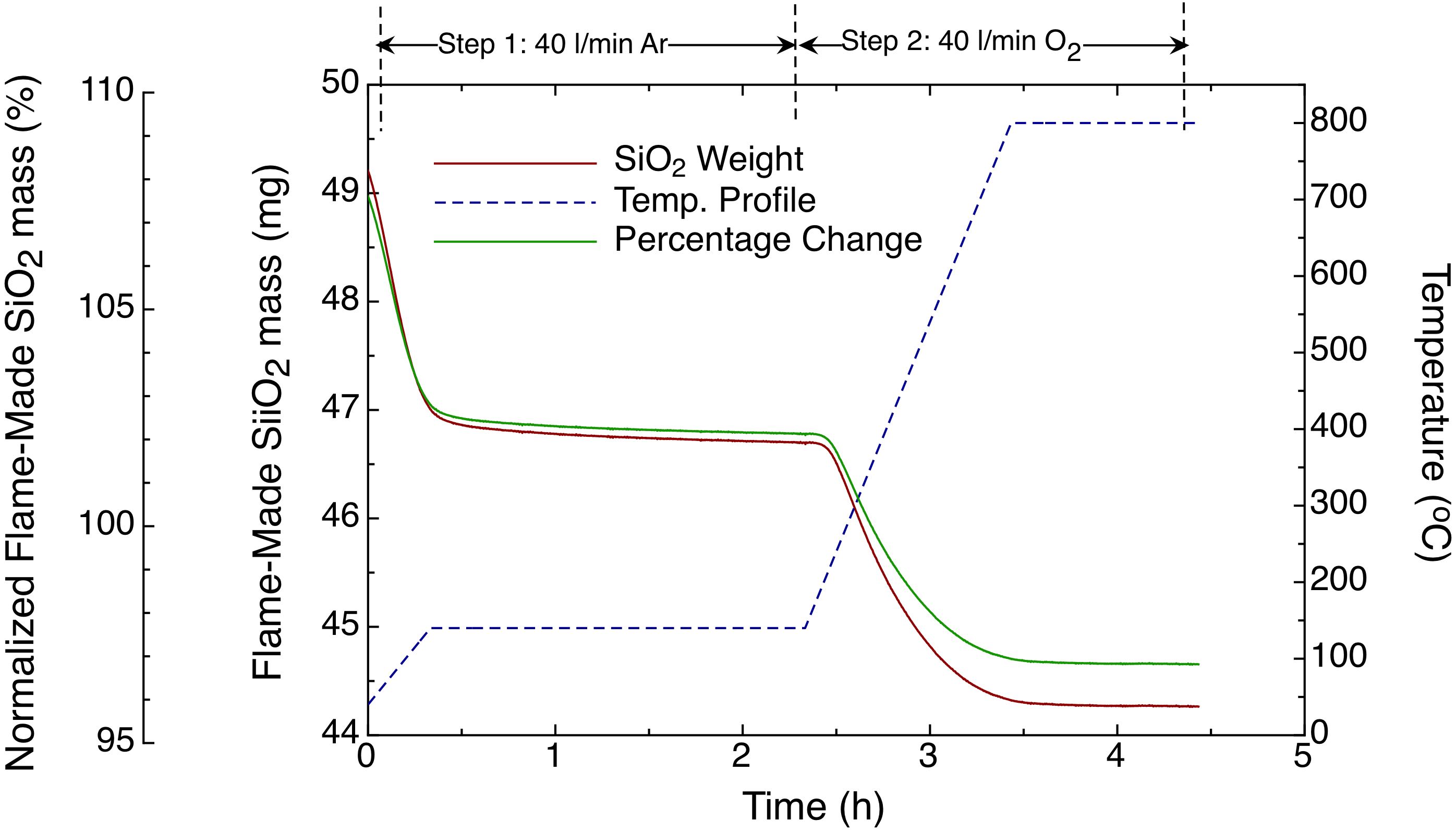


**Figure S1:** TGA temperature-time profile (right ordinate, dashed line), corresponding sample mass (left ordinate) of as-produced (solid lines) and the mass loss normalized to the mass at the end of Step 1.

**
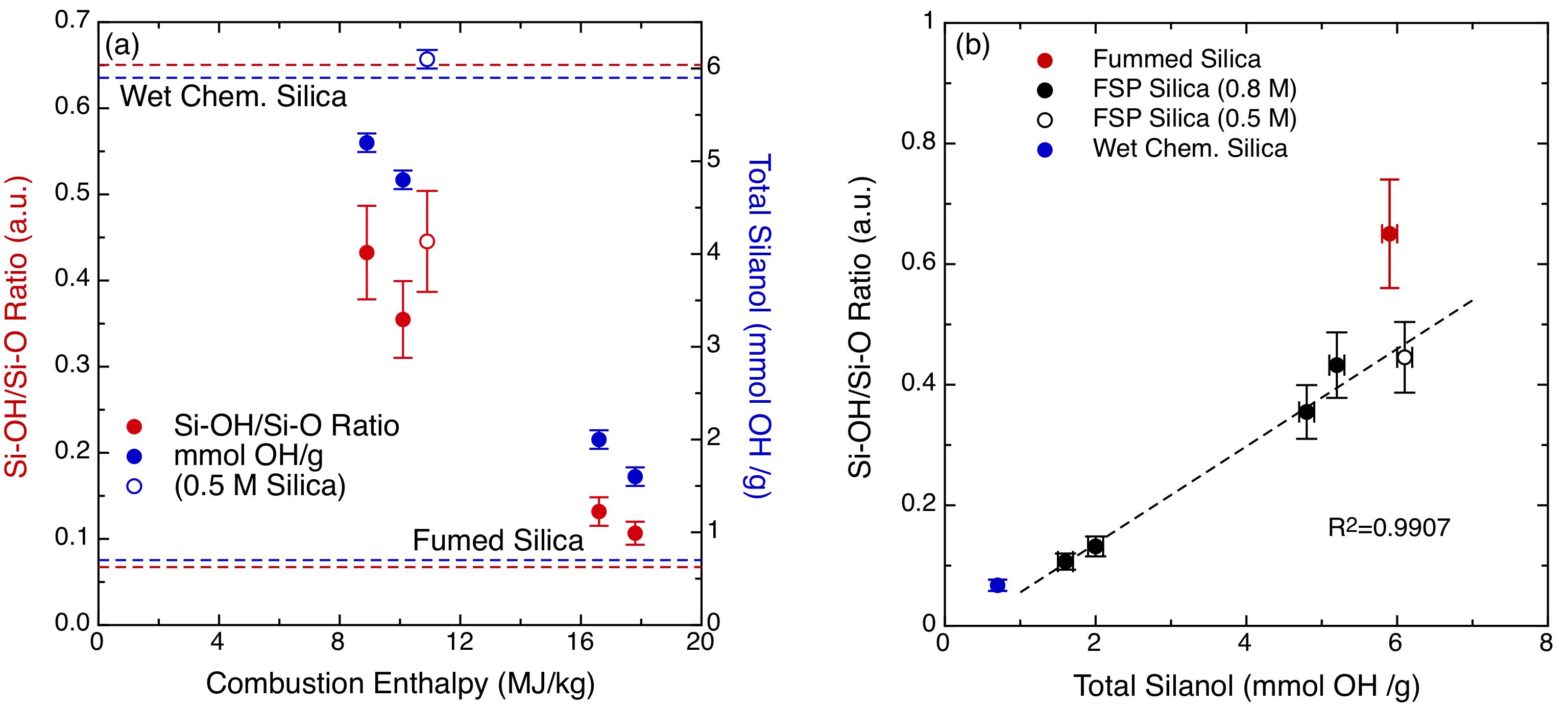
**

**Figure S2:** XPS analysis. (a) Si-OH/O-Si-O ratio and total silanol content varying as a function of the combustion enthalpy. (b) Si-OH/O-Si-O ratio as a function of the total silanol.

**
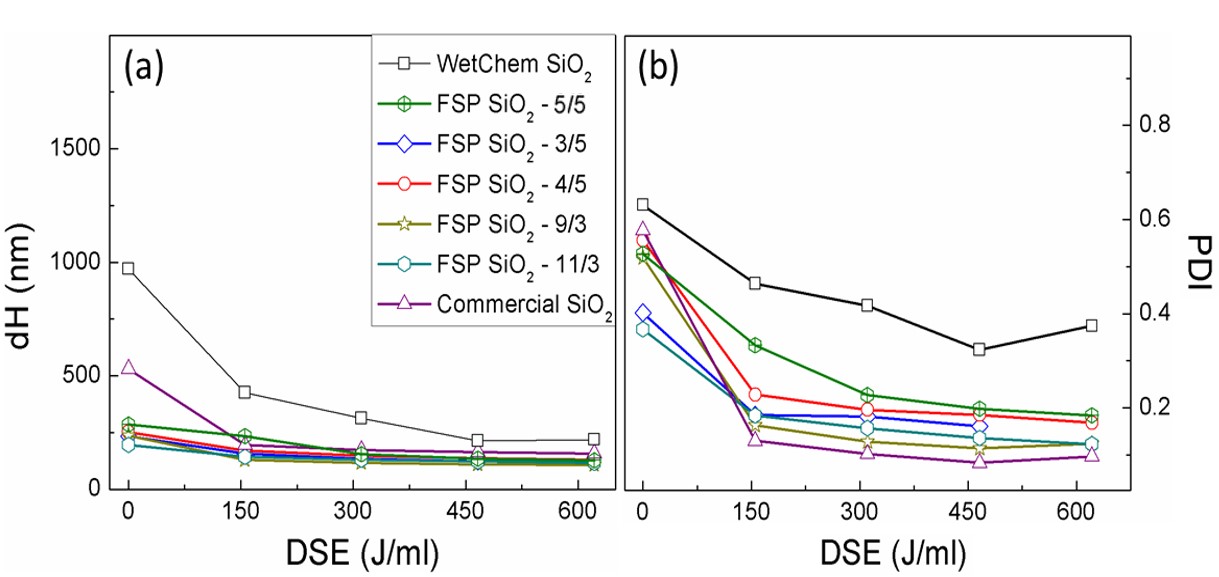
**

**Figure S3:** Determining the critical delivered sonication energy of SiO_2_ NPs. (a) Mean hydrodynamic diameter and (b) polydispersity index as a function of dispersion sonication energy of Wetchem SiO_2_ NPs, FSP made SiO_2_ NPs and commercial fumed SiO_2_ NPs in DI H_2_O.


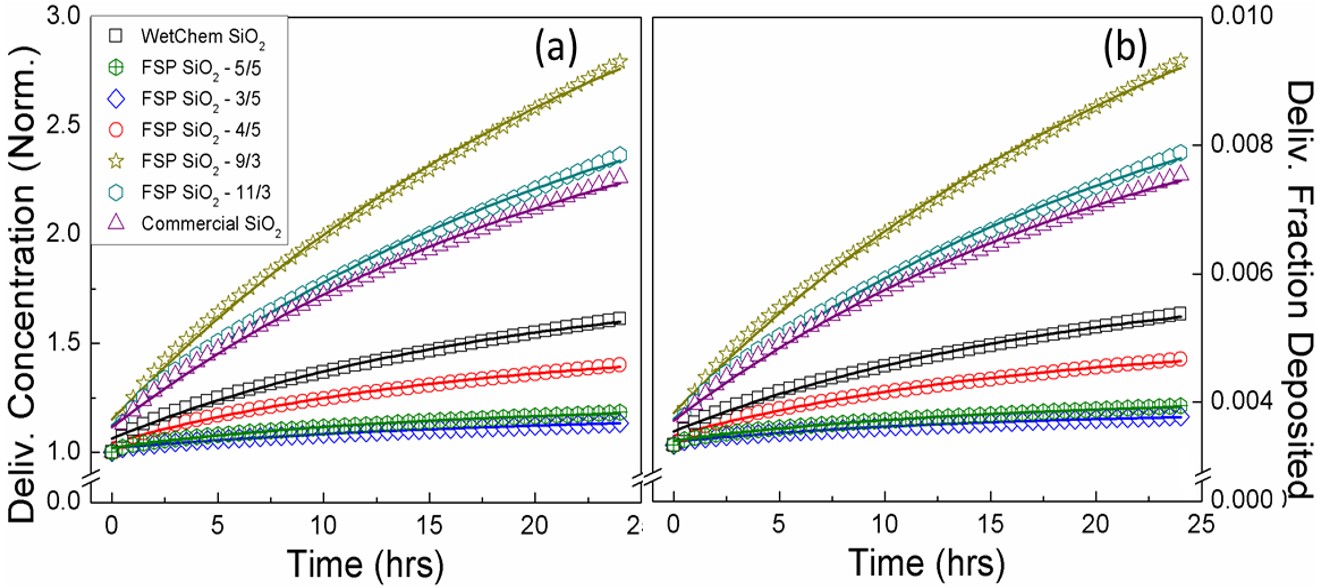


**Figure S4:** Fate and transport modeling results for SiO_2_ NPs. (a) Delivered-to-cell concentration normalized to the administered dose and (b) delivered-to-cell fraction deposited of wet chemistry made silica, FSP made SiO_2_ NPs and commercial fumed SiO_2_ NPs in RPMI + 10% (vol/vol) FBS. Solid lines are the fitting curves obtained using eq. 1 and 2.


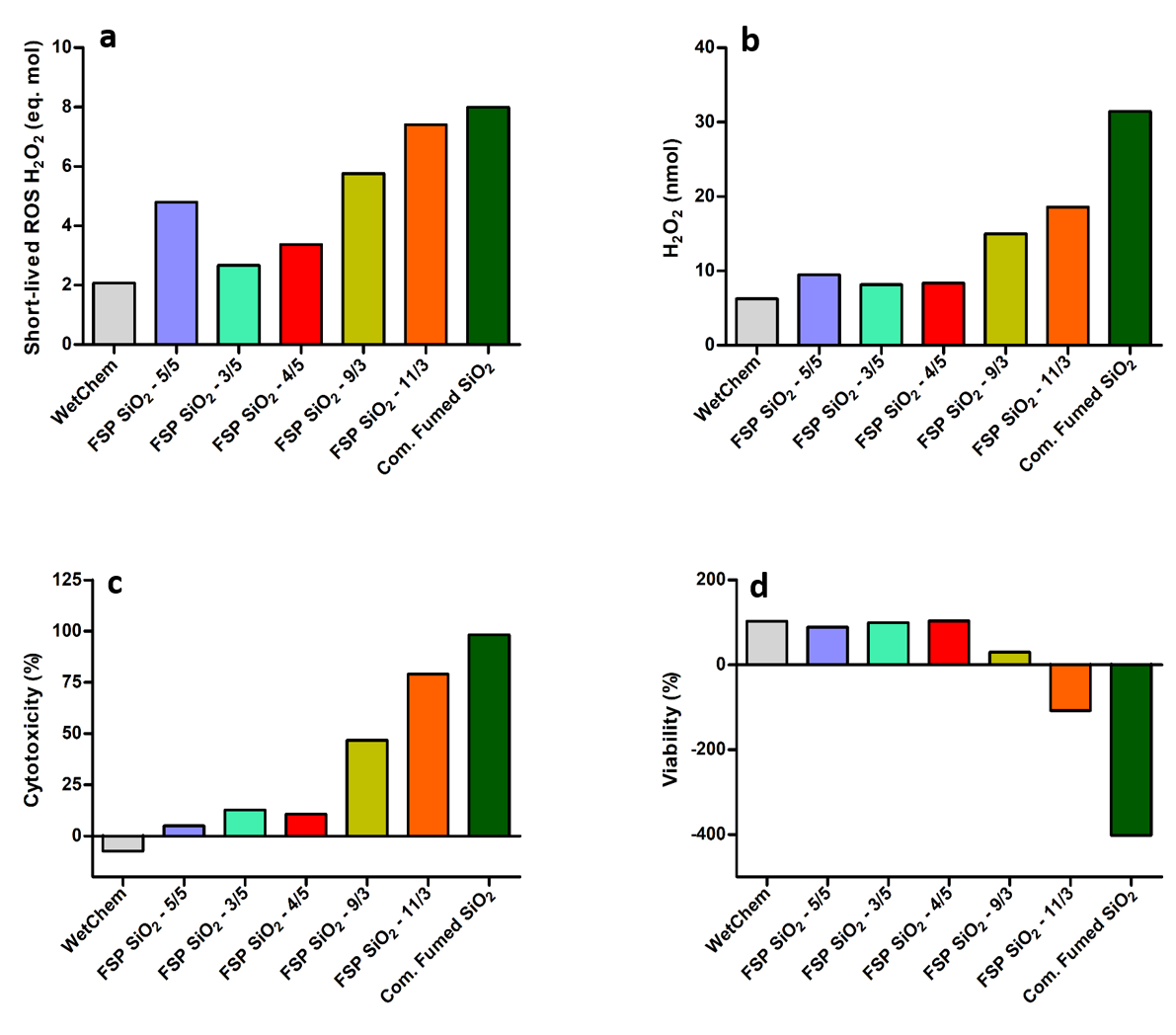


**Figure S5**: Importance of other modulators in silica NPs effect analyzing RAW264.7 cells. (a, b) short-lived ROS and H_2_O_2_ produced by the different SiO_2_ NPs at a fixed value of silanol content of 150 nmol. (c) Cytotoxicity of different SiO_2_ NPs at a fixed value of delivered silanol per cell area of 1 x 10^14^ #/cm^2^. (d) Viability of different SiO_2_ NPs at a fixed value of delivered silanol per cell area of 1.5 x 10^14^ #/cm^2^.


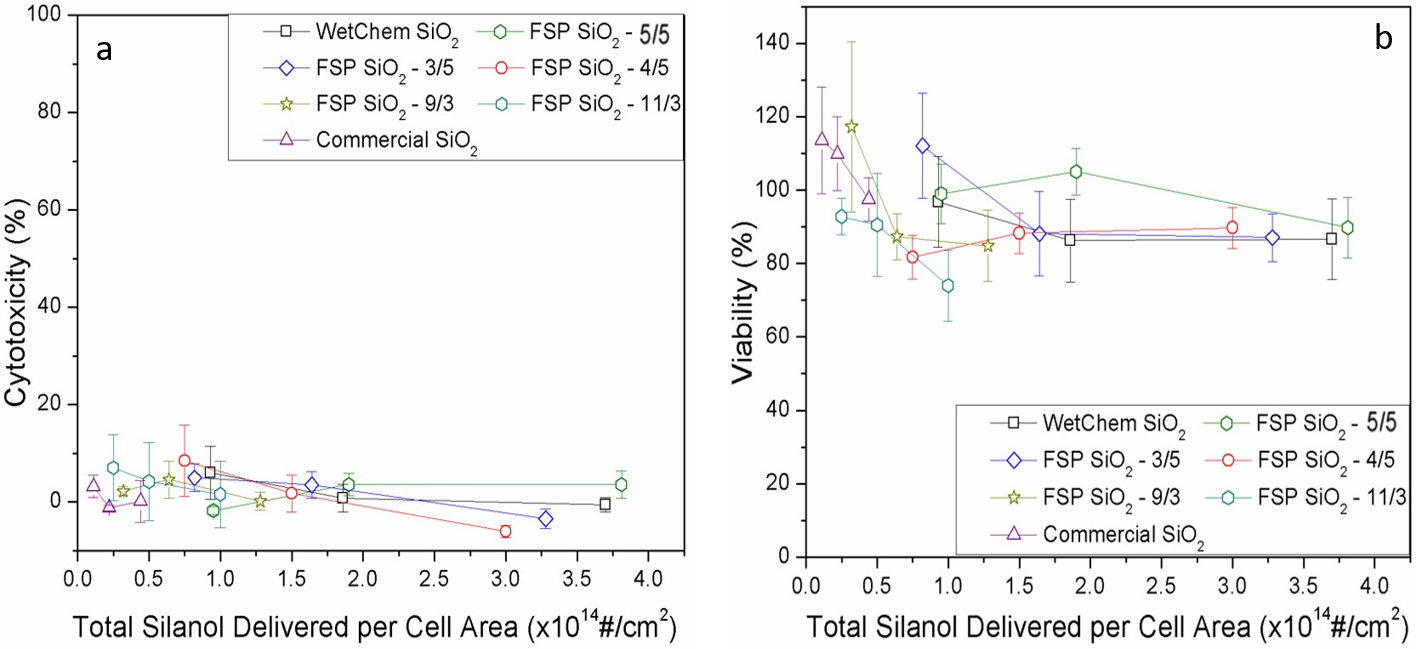


**Fig. S6**. Cytotoxicity (a) and Viability (PrestoBlue assay) (b) measured in SAEC cells. The data represented as function of total silanol delivered per cell area for the three delivered doses used. Data represent an average of three independent experiments performed in triplicate.


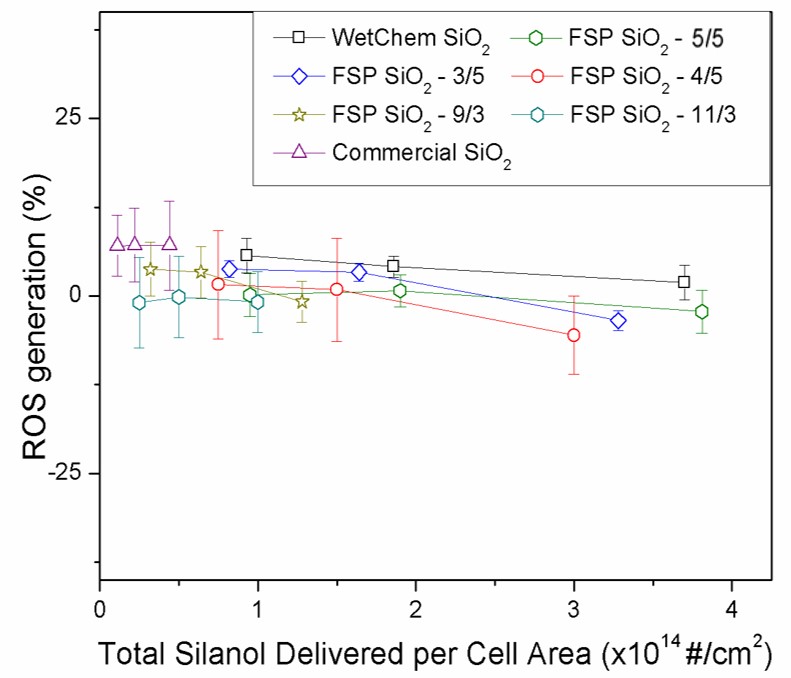


**Fig. S7**. ROS generation as a measure of oxidative damage (CellROX Green assay) in SAEC cells. After 24-hour treatment, ROS generation was measured and data represented as function of total silanol delivered per cell area for the three delivered doses used. Data represent an average of three independent experiments performed in triplicate.


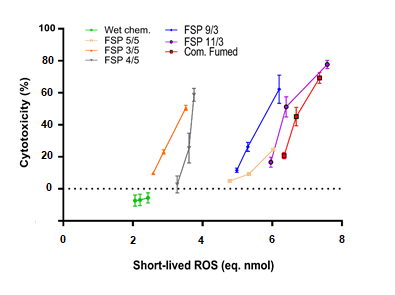


**Fig. S8**. Cytotoxicity measured in RAW264.7 cells. The data is represented as function of short life ROS-H_2_O_2_ eq. nmol. Data represent an average of three independent experiments performed in triplicate.

**Table S1**. Mean values of the parameters obtained for suspension preparation and colloidal characterization of wet chemistry made silica, FSP made silicas and commercial fumed silica in H_2_O and RPMI + 10% (vol/vol) FBS.

| **Silica type** | **DSE_cr_**  **(J/ml)** | **Media** | **Colloidal Properties** | | | | | |
| --- | --- | --- | --- | --- | --- | --- | --- | --- |
|  |  |  | **d_H_ (nm)** | | **PDI** | **ζ (mV)** | **σ (mS/cm)** | **ρ_eff_ (g/cc)** |
| WetChem SiO_2_ | 380 | DI Water | 207.7±26.4 | | 0.57±0.05 | -23.6±4.1 | 0.02±0.00 |  |
|  |  | RPMI/10%/FBS | t=0h  t=24h | 326.8±18.2  228.6±19.5 | 0.52±0.05 | -11.7±0.3 | 10.50±0.10 | 1.31±0.07 |
|  |  |  |  |  | 0.55±0.12 | -12.2±0.9 | 10.40±0.17 |  |
| FSP SiO_2_ -5/5 | 161 | DI Water | 135.0±1.4 | | 0.20±0.01 | -16.2±1.9 | 0.03±0.00 |  |
|  |  | RPMI/10%/FBS | t=0h | 236.2±60.2 | 0.32±0.01 | -11.3±2.0 | 10.30±2.12 | 1.13±0.01 |
|  |  |  | t=24h | 269.0±19.5 | 0.33±0.04 | -12.0±0.1 | 13.61±0.15 |  |
| FSP SiO_2_-3/5 | 200 | DI Water | 137.6±0.7 | | 0.18±0.010.21±0.05  0.22±0.05 | -25.1±3.7  -12.6±3.1  -14.4±0.5 | 0.01±0.00  9.51±0.77  10.60±0.23 |  |
|  |  | RPMI/10%/FBS | t=0h | 228.6±9.2 |  |  |  | 1.12±0.01 |
|  |  |  | t=24h | 196.9±1.8 |  |  |  |  |
| FSP SiO_2_-4/5 | 220 | DI Water | 131.0±0.8 | | 0.16±0.01  0.17±0.01  0.28±0.13 | -15.3±1.2  -12.9±1.2  -12.5±0.1 | 0.01±0.00  8.91±0.54  8.83±0.13 |  |
|  |  | RPMI/10%/FBS | t=0h | 205.8±3.2 |  |  |  | 1.20±0.02 |
|  |  |  | t=24h | 369.5±42.8 |  |  |  |  |
| FSP SiO_2_-9/3 | 205 | DI Water | 158.4±7.2 | | 0.20±0.02  0.14±0.07  0.23±0.07 | -25.9±2.9  -13.1±0.7  -14.6±0.8 | 0.01±0.01  10.80±0.40  10.20±0.11 |  |
|  |  | RPMI/10%/FBS | t=0h | 221.0±3.6 |  |  |  | 1.18±0.02 |
|  |  |  | t=24h | 291.9±19.7 |  |  |  |  |
| FSP SiO_2_-11/3 | 190 | DI Water | 130.8±3.8 | | 0.15±0.02  0.21±0.04  0.34±0.25 | -18.2±1.9  -12.5±0.4  -11.5±0.3 | 0.01±0.00  10.70±0.38  10.20±0.10 |  |
|  |  | RPMI/10%/FBS | t=0h | 218.3±8.3 |  |  |  | 1.25±0.01 |
|  |  |  | t=24h | 227.2±75.1 |  |  |  |  |
| Commercial fumed SiO_2_ | 195 | DI Water | 183.5±1.5 | | 0.12±0.01  0.17±0.03  0.19±0.03 | -23.1±4.6  -11.2±0.7  -5.2±0.9 | 0.01±0.00  10.50±0.32  10.50±0.60 |  |
|  |  | RPMI/10%/FBS | t=0h | 249.3±1.4 |  |  |  | 1.19±0.01 |
|  |  |  | t=24h | 252.5±2.9 |  |  |  |  |

FSP, flame spray pyrolysis; DSEcr, critical delivered sonication energy; d_H_, in water is determined from the DLS intensity-weighted size distribution, whereas in medium the DLS volume-weighted size distribution; PDI, polydispersity index; ζ, zeta potential; σ, specific conductance; ρ_eff_, effective density.

**Table S2:** The short-lived ROS and H_2_O_2_ generated from seven types of silica over the 10-100 µg/mL range. Values have been corrected for sonication and background oxidation of Trolox.

| Dose (µg/mL) | Silica type – All ROS and H2O2 values are in nmol/mL | | | | | | | | | | | | | |
| --- | --- | --- | --- | --- | --- | --- | --- | --- | --- | --- | --- | --- | --- | --- |
|  | **WetChem** | | **FSP 5/5** | | **FSP 3/5** | | **FSP 4/5** | | **FSP 9/3** | | **FSP 11/3** | | **Commercial** | |
|  | ROS | H_2_O_2_ | ROS | H_2_O_2_ | ROS | H_2_O_2_ | ROS | H_2_O_2_ | ROS | H_2_O_2_ | ROS | H_2_O_2_ | ROS | H_2_O_2_ |
| 10 | 2.01 | 5.78 | 4.08 | 8.52 | 2.49 | 7.03 | 2.95 | 7.23 | 4.63 | 11.53 | 5.70 | 15.38 | 5.37 | 15.07 |
| 25 | 2.06 | 6.22 | 4.78 | 9.35 | 2.58 | 8.05 | 3.27 | 7.61 | 4.98 | 12.26 | 5.96 | 16.08 | 6.34 | 15.87 |
| 50 | 2.20 | 7.03 | 5.33 | 10.67 | 2.88 | 8.52 | 3.61 | 8.86 | 5.30 | 14.36 | 6.40 | 15.91 | 6.69 | 16.01 |
| 100 | 2.43 | 8.65 | 6.03 | 10.69 | 3.52 | 9.62 | 3.75 | 9.46 | 6.20 | 15.98 | 7.58 | 19.24 | 7.36 | 19.06 |

**Cellular statistical analysis**

| **Tukey's multiple comparisons test** | Mean Diff, | 95,00% CI of diff, | Significant? | Summary | Adjusted P Value |
| --- | --- | --- | --- | --- | --- |
|  |  |  |  |  |  |
| **0.026 µg/cm2** | | | | | |
| Wet chem. vs. FSP 5/5 | -12,29 | -22,89 to -1,678 | Yes | * | 0,0141 |
| Wet chem. vs. FSP 3/5 | -17,13 | -27,73 to -6,519 | Yes | *** | 0,0002 |
| Wet chem. vs. FSP 4/5 | -10,03 | -20,64 to 0,5768 | No | ns | 0,0745 |
| Wet chem. vs. FSP 9/3 | -18,92 | -29,52 to -8,308 | Yes | **** | <0,0001 |
| Wet chem. vs. FSP 11/3 | -23,98 | -34,59 to -13,37 | Yes | **** | <0,0001 |
| Wet chem. vs. Com. Fumed | -28,02 | -38,63 to -17,41 | Yes | **** | <0,0001 |
| FSP 5/5 vs. FSP 3/5 | -4,841 | -15,45 to 5,767 | No | ns | 0,7920 |
| FSP 5/5 vs. FSP 4/5 | 2,255 | -8,353 to 12,86 | No | ns | 0,9942 |
| FSP 5/5 vs. FSP 9/3 | -6,63 | -17,24 to 3,978 | No | ns | 0,4698 |
| FSP 5/5 vs. FSP 11/3 | -11,69 | -22,3 to -1,086 | Yes | * | 0,0224 |
| FSP 5/5 vs. Com. Fumed | -15,73 | -26,34 to -5,126 | Yes | *** | 0,0007 |
| FSP 3/5 vs. FSP 4/5 | 7,096 | -3,512 to 17,7 | No | ns | 0,3882 |
| FSP 3/5 vs. FSP 9/3 | -1,789 | -12,4 to 8,818 | No | ns | 0,9984 |
| FSP 3/5 vs. FSP 11/3 | -6,853 | -17,46 to 3,755 | No | ns | 0,4300 |
| FSP 3/5 vs. Com. Fumed | -10,89 | -21,5 to -0,2848 | Yes | * | 0,0407 |
| FSP 4/5 vs. FSP 9/3 | -8,885 | -19,49 to 1,723 | No | ns | 0,1542 |
| FSP 4/5 vs. FSP 11/3 | -13,95 | -24,56 to -3,341 | Yes | ** | 0,0036 |
| FSP 4/5 vs. Com. Fumed | -17,99 | -28,6 to -7,38 | Yes | **** | <0,0001 |
| FSP 9/3 vs. FSP 11/3 | -5,063 | -15,67 to 5,544 | No | ns | 0,7561 |
| FSP 9/3 vs. Com. Fumed | -9,103 | -19,71 to 1,505 | No | ns | 0,1353 |
| FSP 11/3 vs. Com. Fumed | -4,04 | -14,65 to 6,568 | No | ns | 0,8981 |
|  |  |  |  |  |  |
| **0.052 µg/cm2** | | | | | |
| Wet chem. vs. FSP 5/5 | -16,23 | -26,84 to -5,62 | Yes | *** | 0,0005 |
| Wet chem. vs. FSP 3/5 | -30,02 | -40,63 to -19,42 | Yes | **** | <0,0001 |
| Wet chem. vs. FSP 4/5 | -32,38 | -42,99 to -21,77 | Yes | **** | <0,0001 |
| Wet chem. vs. FSP 9/3 | -33,18 | -43,78 to -22,57 | Yes | **** | <0,0001 |
| Wet chem. vs. FSP 11/3 | -58,23 | -68,84 to -47,62 | Yes | **** | <0,0001 |
| Wet chem. vs. Com. Fumed | -52,14 | -62,75 to -41,53 | Yes | **** | <0,0001 |
| FSP 5/5 vs. FSP 3/5 | -13,8 | -24,4 to -3,188 | Yes | ** | 0,0040 |
| FSP 5/5 vs. FSP 4/5 | -16,15 | -26,76 to -5,545 | Yes | *** | 0,0005 |
| FSP 5/5 vs. FSP 9/3 | -16,95 | -27,56 to -6,341 | Yes | *** | 0,0002 |
| FSP 5/5 vs. FSP 11/3 | -42 | -52,61 to -31,4 | Yes | **** | <0,0001 |
| FSP 5/5 vs. Com. Fumed | -35,91 | -46,52 to -25,3 | Yes | **** | <0,0001 |
| FSP 3/5 vs. FSP 4/5 | -2,357 | -12,96 to 8,251 | No | ns | 0,9926 |
| FSP 3/5 vs. FSP 9/3 | -3,153 | -13,76 to 7,455 | No | ns | 0,9672 |
| FSP 3/5 vs. FSP 11/3 | -28,21 | -38,82 to -17,6 | Yes | **** | <0,0001 |
| FSP 3/5 vs. Com. Fumed | -22,12 | -32,72 to -11,51 | Yes | **** | <0,0001 |
| FSP 4/5 vs. FSP 9/3 | -0,7958 | -11,4 to 9,812 | No | ns | >0,9999 |
| FSP 4/5 vs. FSP 11/3 | -25,85 | -36,46 to -15,24 | Yes | **** | <0,0001 |
| FSP 4/5 vs. Com. Fumed | -19,76 | -30,37 to -9,152 | Yes | **** | <0,0001 |
| FSP 9/3 vs. FSP 11/3 | -25,05 | -35,66 to -14,45 | Yes | **** | <0,0001 |
| FSP 9/3 vs. Com. Fumed | -18,96 | -29,57 to -8,356 | Yes | **** | <0,0001 |
| FSP 11/3 vs. Com. Fumed | 6,091 | -4,517 to 16,7 | No | ns | 0,5701 |
|  |  |  |  |  |  |
| **0.104 µg/cm2** | | | | | |
| Wet chem. vs. FSP 5/5 | -30,27 | -40,87 to -19,66 | Yes | **** | <0,0001 |
| Wet chem. vs. FSP 3/5 | -56,32 | -66,92 to -45,71 | Yes | **** | <0,0001 |
| Wet chem. vs. FSP 4/5 | -64,39 | -75 to -53,78 | Yes | **** | <0,0001 |
| Wet chem. vs. FSP 9/3 | -67,89 | -78,5 to -57,28 | Yes | **** | <0,0001 |
| Wet chem. vs. FSP 11/3 | -83,41 | -94,02 to -72,8 | Yes | **** | <0,0001 |
| Wet chem. vs. Com. Fumed | -75,07 | -85,68 to -64,46 | Yes | **** | <0,0001 |
| FSP 5/5 vs. FSP 3/5 | -26,05 | -36,66 to -15,44 | Yes | **** | <0,0001 |
| FSP 5/5 vs. FSP 4/5 | -34,13 | -44,73 to -23,52 | Yes | **** | <0,0001 |
| FSP 5/5 vs. FSP 9/3 | -37,62 | -48,23 to -27,01 | Yes | **** | <0,0001 |
| FSP 5/5 vs. FSP 11/3 | -53,15 | -63,75 to -42,54 | Yes | **** | <0,0001 |
| FSP 5/5 vs. Com. Fumed | -44,81 | -55,42 to -34,2 | Yes | **** | <0,0001 |
| FSP 3/5 vs. FSP 4/5 | -8,075 | -18,68 to 2,533 | No | ns | 0,2426 |
| FSP 3/5 vs. FSP 9/3 | -11,57 | -22,18 to -0,9642 | Yes | * | 0,0246 |
| FSP 3/5 vs. FSP 11/3 | -27,1 | -37,7 to -16,49 | Yes | **** | <0,0001 |
| FSP 3/5 vs. Com. Fumed | -18,76 | -29,36 to -8,149 | Yes | **** | <0,0001 |
| FSP 4/5 vs. FSP 9/3 | -3,498 | -14,11 to 7,11 | No | ns | 0,9463 |
| FSP 4/5 vs. FSP 11/3 | -19,02 | -29,63 to -8,414 | Yes | **** | <0,0001 |
| FSP 4/5 vs. Com. Fumed | -10,68 | -21,29 to -0,0746 | Yes | * | 0,0474 |
| FSP 9/3 vs. FSP 11/3 | -15,52 | -26,13 to -4,916 | Yes | *** | 0,0009 |
| FSP 9/3 vs. Com. Fumed | -7,185 | -17,79 to 3,423 | No | ns | 0,3733 |
| FSP 11/3 vs. Com. Fumed | 8,339 | -2,269 to 18,95 | No | ns | 0,2105 |
